# Small RNA or oligonucleotide drugs and challenges in evaluating drug-drug interactions

**DOI:** 10.3389/fphar.2025.1720361

**Published:** 2025-11-27

**Authors:** Joseph M. Cronin, Ai-Ming Yu

**Affiliations:** Department of Biochemistry and Molecular Medicine, University of California at Davis, School of Medicine, Sacramento, CA, United States

**Keywords:** oligonucelotide, RNA drug, drug interaction, PK/PD (pharmacokinetics/pharmacodynamics), ADME, RNAi-RNA interference, ASO-antisense oligonucleotides, siRNA-small interfering RNA

## Abstract

Small RNA or oligonucleotide therapeutics represent a unique modality outside the traditional treatment paradigm of small molecule and protein-based drugs that have historically only targeted a small fraction of the proteome. Innovations in the structural design and chemical modification have been invaluable for recent oligonucleotide therapeutics, greatly improving their biological stability, intracellular delivery, and targeting. Widespread adoption of these strategies has further enabled the application of oligonucleotides as viable drugs and expanded the class of RNA therapeutics, with thirteen antisense oligonucleotides (ASOs) (fomiversen, mipomersen, nusinersen, inotersen, eteplirsen, golodirsen, casimersen, viltolarsen, tofersen, eplontersen, olezarsen, and donidalorsen), seven small interfering RNAs (siRNAs) (patisiran, givosiran, lumasiran, inclisiran, vutrisiran, nedosiran, and fitusiran), and two aptamers (pegaptanib and avacincaptad pegol) that have been approved by the United States Food and Drug Administration (FDA). RNA therapeutics have expanded the druggable space and provide a novel treatment strategy, they do not fit within the framework of our current methodology in evaluating risk of drug-drug interactions (DDIs) and assessing pharmacokinetic/pharmacodynamic (PK/PD) relationships. This article provides an overview of FDA-approved oligonucleotide therapies, emphasizing chemical modifications, molecular targets for mechanistic actions, and available ADME and PK/PD properties, followed by the discussion of critical needs for risk assessment strategies suited for this unique modality that focuses on possible DDIs with concomitant drugs. The latter may involve direct competition for the endogenous RNA interference machinery to alter ADME or relevant PD gene expression, rather than uncommon binding or interactions with drug-metabolizing enzymes or transporters found and recommended for small molecule drugs.

## 1 Introduction

Conventional therapeutic strategies centered around protein-targeted drugs have been constrained by our limited understanding of RNAs as simply a set of messengers that relay the genetic code into functional protein. With only a small percentage of messenger RNA (mRNA) encoded proteins being considered druggable, research into protein-targeted therapies was limited to a fraction of the proteome. The discovery of many functional RNA species that did not encode proteins provided a perspective of protein expression and RNA function that was much more complex, but quickly showed therapeutic potential when a single-stranded oligonucleotide was found to target mRNA and control gene expression (Sutcliffe, 1978). This continued with the discovery of small non-coding microRNAs (miRNAs or miRs), which were naturally expressed within *Caenorhabditis elegans* to elicit post-transcriptional gene regulation (PTGR) of target mRNA via antisense interaction (Lee et al., 1993; Wightman et al., 1993). Soon after, exogenous double-stranded RNAs showed regulation of complementary mRNA, a mechanism referred to as RNA interference (RNAi) that set the stage for future therapeutic use of small interfering RNAs (siRNAs) (Fire et al., 1998; Zamore et al., 2000).

These discoveries gave rise to RNA-based therapeutics, a novel class of therapies that are fundamentally distinct from traditional small molecules and protein biologics. This class encompasses a diverse set of therapeutic strategies including aptamers, antisense oligonucleotides (ASOs), and siRNAs, which modulate protein expression or activity of their specific targets (Yu and Tu, 2022; Jin et al., 2025). These therapies exhibit unique chemical structures and diverse mechanisms of action that are critically different from small molecule and biologic protein drugs. RNA therapeutics’ unique targeting and mechanistic actions expand the range of druggable molecules within the genome, but they also differ in their pharmacokinetic (PK) properties compared to well-understood small molecule and protein-based drugs. Oligonucleotide drugs are naturally unstable in biological systems, being readily degraded by natural human endo- and exonucleases that cleave phosphodiester bonds at unique locations within the molecule (Kovacevic et al., 2018; Post et al., 2019; Li et al., 2021; Zhang et al., 2025). Additionally, the charged backbone, hydrophilic nature, and large size of many oligonucleotide drugs prevent them from crossing cell membranes, posing significant delivery challenges, a major concern affecting clinical drug administration, distribution, metabolism, and excretion (ADME) processes. These challenges are commonly addressed by chemical modification of the phosphodiester linkages, ribose sugar rings, and nucleobases, as well as through the conjugation of large polymers or ligands. These modifications confer nuclease resistance, improve oligo delivery and PK, and enable specific targeting to hepatocytes through the use of ligands like *N*-acetylgalactosamine (GalNAc) (Wang et al., 2019; Li et al., 2021; Jeon et al., 2022; McDougall et al., 2022).

Distinct from small molecule and protein drugs, oligonucleotides are not major substrates of cytochrome P450 enzymes but subject to cleavage by various nucleases and hydrolases, nor are they typical substrates for efflux transporters, hepatic uptake transporters, or renal uptake transporters but subject to endocytosis or receptor mediated uptake, and thus exhibit nontraditional PK properties. Further, systemic PK parameters of RNA therapies do not always reflect target tissue distribution and are not often reflective of pharmacodynamic (PD) outcomes, highlighting the need for additional pharmacodynamic endpoints in multiple-dose studies such as target mRNA/protein or other relevant PD biomarkers (U.S. Food and Drug Administration, 2024b).

RNA therapeutics’ sequence driven specificity offers novel therapeutic opportunities, but they also present unique challenges in evaluating risk of drug-drug interactions (DDIs) and adverse events, from both PK and PD perspectives. With these factors in mind, the U.S. Food and Drug Administration (FDA) recommends several key risk evaluations including assessing potential for QTc interval prolongation, as well as assessing the impact of hepatic and renal impairment on pharmacodynamic endpoints and biomarkers independent of PK, with participants across a range of organ function. The distinct structures, non-natural chemical modifications, unique mechanistic actions, and nebulous PK/PD relationships of oligonucleotide therapeutics underscore the needs for risk assessment strategies uniquely tailored to this modality, including immunogenicity, on/off-target effects, and PD-based DDI with drugs or endogenous RNA that share mechanistic action or pathways. Indeed, RNAi or ASO drugs would likely compete with the genome-derived miRNAs for the intrinsic RNAi components and subsequently, alter the PTGR of ADME or related PD genes and then lead to possible DDIs (Yu, 2007; Yu and Pan, 2012; Jilek et al., 2017; Traber et al., 2025).

This review will first introduce the U.S. Food and Drug Administration (FDA) approved oligonucleotide drugs, their molecular targets, mechanistic actions, therapeutic indications, known ADME/PK properties, and discuss the challenges in the evaluation of DDIs for RNA therapeutics (U.S. Food and Drug Administration, 2024b).

## 2 Common mechanistic actions of RNA therapeutics

**FIGURE 1:**
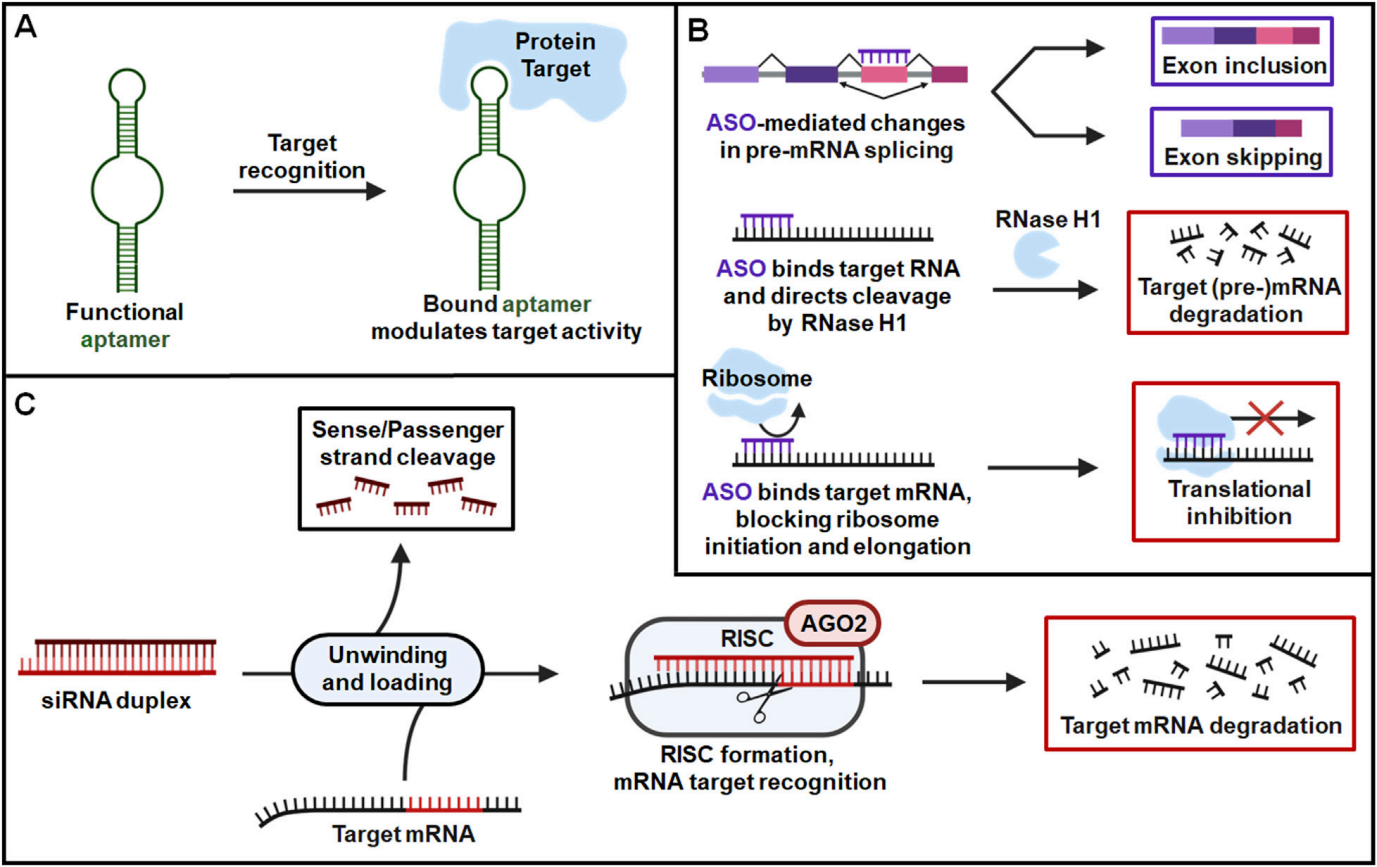
General pharmacological actions of FDA-approved RNA therapeutics. **(A)** Aptamers are single-stranded oligonucleotides that fold into unique structures to specifically bind their protein targets and modulate their activities. **(B)** ASOs are short, single-stranded oligonucleotides that target specific RNA sequences via Watson-Crick base pairing and alter expression of their corresponding proteins. ASOs can modulate target expression by several distinct mechanisms such as alternative splicing of pre-mRNA, induction of RNase H1-dependent cleavage of target RNA, and inhibition of target RNA translation through steric blockage of ribosome interaction. **(C)** SiRNAs are double stranded RNAs containing guide/antisense strands designed to target specific mRNA sequences by (near-)perfect complementary base pairing with target transcripts. After the siRNA is unwounded, the antisense strand is uploaded into the RISC where it identifies and cleaves complementary target mRNA sequence to modulate protein outcome.

Fundamental scientific research uncovering the diverse forms and functions of natural RNAs beyond encoding protein has provided valuable inspiration in the design and application of RNA therapeutics. RNA has since emerged as a versatile treatment modality, employing several different mechanisms of action across FDA-approved therapies. While there are many exciting therapeutic applications of different forms of RNAs under clinical and preclinical development, this review will focus on the FDA-approved aptamers, ASOs, siRNAs (Figure 1).

Aptamers are single-stranded oligonucleotides optimized for high-affinity binding to a specific protein sequence, commonly acting as inhibitors or antagonists of their target to reduce protein activities or functions (Yu et al., 2020) (Figure 1). Aptamers that function as agonists and allosteric modulators of their target have also been developed, although none have yet reached the clinic (Yunn et al., 2022; Kim et al., 2025). Aptamer drugs assume complex tertiary structures that allow binding of their target with a high degree of specificity. Oligonucleotide sequences form a diverse range of structural motifs that are utilized in aptamer design to target a variety of molecules beyond just proteins, including nucleic acids, small molecules, and heavy metals (Ruscito and DeRosa, 2016; Yu et al., 2020). While other strategies are promising, both FDA-approved aptamer therapies function through protein targeting (Figure 1) (U.S. Food and Drug Administration, 2011; 2025d).

ASOs are short, single-stranded oligonucleotides that engage with specific RNA sequences through Watson-Crick base pairing (Yu et al., 2020; Yu and Tu, 2022). FDA-approved ASO therapies bind target mRNA and regulate protein expression through several possible mechanisms, including cleavage of the target transcript by RNase H, and inhibition of its translation through steric blockage of ribosome binding. ASO therapies can also act through alternative splicing of pre-mRNA to promote inclusion or exclusion of specific exons, altering the identity of the corresponding protein (Figure 1). These mechanisms encompass all FDA-approved ASOs, with other types of remedies, such as ASO against disease-associated miRNAs, showing limited success in the clinic (Gebert et al., 2014; Martino et al., 2025).

SiRNAs are double stranded RNA molecules that form complementary base pairings with target mRNA transcripts, modulating the levels of corresponding proteins through RNAi pathway (Yu et al., 2020; Traber and Yu, 2023; Traber and Yu, 2024). SiRNA therapies contain an antisense/guide strand designed to recognize a sequence within the coding region or 3′untranslated region (UTR) of a target transcript, where they bind with (near-) perfect complementarity. Administered siRNA duplexes within the cytoplasm are unwound and the antisense/guide strand is preferentially loaded into the RNA induced silencing complex (RISC). The guide identifies complementary mRNA sequences to direct RISC-mediated cleavage of target transcripts, leading to the reduction of protein levels (Figure 1). The therapeutic action of siRNA cannot happen without the key machinery that comprises the RISC, with Argonaute (AGO) protein serving as a critical and necessary component. The four AGO isoforms in humans serve similar functions in RNAi, but only AGO2 possesses the catalytic activity needed for nucleolytic cleavage of RISC-bound target mRNA. This is key for siRNA drugs as they possess highly complementary guide strands, form ideal interactions for mRNA cleavage, and preferentially engage with AGO2/RISC. By contrast, endogenous miRNAs acting through RNAi machinery typically binds via the seed region spanning 2-7 nt and can interact with AGO1-4/RISC, forming shorter complementary interactions that are less specific but allowing targeting of multiple unique mRNA transcripts. This seed-mediated target binding by miRNA does not rapidly cleave the mRNA but instead inhibits its translation and sometimes promotes mRNA degradation. Despite this distinction, siRNA and miRNA may compete for key parts of the RISC, with AGO2 serving as a major example. Later in this review, we will discuss the unique challenges this poses in evaluating DDI risk and provide perspective for the future study and intelligent design of RNAi therapeutics.

## 3 Aptamer drugs

Pegaptanib (Macugen) was the first FDA-approved aptamer drug and the first therapy to target vascular endothelial growth factor (VEGF), being approved in 2004 for the treatment of age-related macular degeneration (AMD) (Table 1) (Gragoudas et al., 2004; U.S. Food and Drug Administration, 2011; Yu et al., 2020). Pegaptanib treats the neovascular (wet) form of AMD, where abnormal blood vessel growth causes loss of vision. The 28-nucleotide RNA sequence of pegaptanib specifically targets the heparin binding domain of extracellular VEGF, preventing efficient activation of VEGF receptors and inhibiting abnormal blood vessel growth within the eye to halt vision loss. Pegaptanib’s tertiary structure provides isoform-selective targeting of VEGF, binding the extracellular isoform most relevant to AMD progression (VEGF-165) while sparing other biologically active isoforms important in heathy function (VEGF-121) (Ruckman et al., 1998; Lee et al., 2005; Kim, 2007). Pegaptanib proved effective in two concurrent clinical trials, where 70% of patients lost less than 15 letters of visual acuity compared to 55% in control groups over 54 weeks. This was also reflected in reduced risk of severe vision depreciation (loss of 30 letters of visual acuity or more) being twice as likely in the sham-injection group compared to pegaptanib treatment group (Gragoudas et al., 2004). Significant benefit was evident across all doses assessed (0.3 mg, 1 mg, 3 mg).

**TABLE 1:** FDA-approved aptamer drugs.

Aptamer drug *RoA*	Chemistry	Target protein	Indication	Year approved
Pegaptanib (Macugen®) *IVT*	PEGylated 28-nt RNA aptamer; all PO (∼) linkages; NaCl/NaPO_4_ solution for *IVT* injection; MW = ∼50 kDa (sodium salt bound to two 20 kDa PEG moieties) R_1_ ― 5′-Cf ∼ Gm ∼ Gm ∼ A ∼ A ∼ Uf ∼ Cf ∼ Am ∼ Gm ∼ Uf ∼ Gm ∼ Am ∼ Am ∼ Uf ∼ Gm ∼ Cf ∼ Uf ∼ Uf ∼ Am ∼ Uf ∼ Am ∼ Cf ∼ Am ∼ Uf ∼ Cf ∼ Cf ∼ Gm∼(3′–3′)dT-5’ 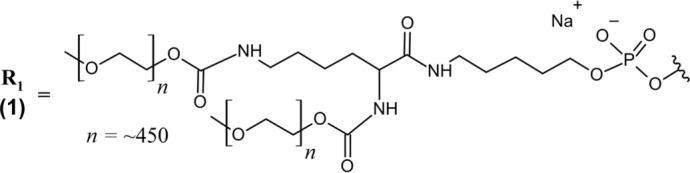	VEGF	AMD	2004
Avacincaptad pegol (Izervay^™^) *IVT*	PEGylated 39-nt RNA aptamer; all PO (∼) linkages; NaCl/NaPO_4_ solution for *IVT* injection; MW = 56 kDa (free acid bound to one 43 kDa PEG moiety) R_2_ ― 5′-Cf ∼ Gm ∼ Cf ∼ Cf ∼ G ∼ Cf ∼ Gm ∼ Gm ∼ Uf ∼ Cf ∼ Uf ∼ Cf ∼ Am ∼ Gm ∼ Gm ∼ Cf ∼ G ∼ Cf ∼ Uf ∼ Gm ∼ Am ∼ Gm ∼ Uf ∼ Cf ∼ Uf ∼ Gm ∼ Am ∼ Gm ∼ Uf ∼ Uf ∼ Uf ∼ A ∼ Cf ∼ Cf ∼ Uf ∼ Gm ∼ Cf ∼ Gm∼(3′–3′)dT-5′ 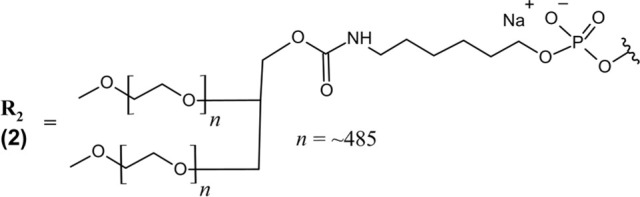	Complement protein C5	GA/AMD	2023

Abbreviations: (3′–3′)dT, inverted thymidine; A, adenosine; Am, 2′-O-methyladenosine; AMD, age-related macular degeneration; C, cytidine; C5, complement component 5; Cf, 2′-fluorocytidine; d, deoxyribonucleoside; G, guanosine; GA, geographic atrophy; Gm, 2′-O-methylguanosine; IVT, intravitreal; PEG, polyethylene glycol; PO (∼), phosphodiester linkage; RoA, route of administration; T, thymidine; U, uridine; Uf, 2′-fluorouridine; VEGF, vascular endothelial growth factor. In the absence of any other label, 2′-deoxynucleosides are assumed.

Pegaptanib contains extensive chemical modifications that protect it from nuclease degradation, improve its PK properties, and improve target binding. All pyrimidines are 2′-deoxy-2′-fluoro nucleosides, while all purine nucleosides contain 2′-*O*-methyl modifications with the exception of two adenosines (positions 4 and 5) (Table 1) (U.S. Food and Drug Administration, 2011). Additionally, the 3′terminal end is an inverted deoxythymidine bound through a 3′–3′ phosphodiester linkage, further improving the aptamer’s metabolic stability. The 5′end of pegaptanib is conjugated to two 20 kDa polyethylene glycol (PEG) moieties via aminohexyl linkage, greatly increasing its size to support a long half-life within the vitreous humor and limit its systemic clearance by renal filtration (Kovacevic et al., 2018; Adachi and Nakamura, 2019). Renal clearance was the major route of elimination identified for pegaptanib, and patients with impaired renal function were assessed in PK studies (Drolet et al., 2000; Basile et al., 2012). However, these patients did not require dose adjustment–likely because of pegaptanib’s slow absorption to the plasma following intravitreal (IVT) injection.

Many years later in 2023, Avacincaptad pegol (Izervay) would become the second aptamer drug approved by the FDA, indicated for the treatment of geographic atrophy (GA) secondary to AMD (Table 1) (U.S. Food and Drug Administration, 2025d). GA is the advanced stage of dry AMD, where chronic inflammation and oxidative stress lead to the irreversible loss of retinal cells and eventually the loss of central vision. Avacincaptad pegol slows the progression of GA by inhibiting the complement system, an inflammatory signaling cascade of the immune system (Danzig and Loewenstein, 2024b). Avacincaptad pegol specifically binds to complement protein C5 and prevents its cleavage to C5a and C5b, lessening subsequent inflammatory signaling that drives cell death and GA progression (Biesecker et al., 1999; Nozaki et al., 2006; Jaffe et al., 2021). In the GATHER1/2 trials, 3.4% of treated patients exhibited persistent loss of visual acuity (>15 letters) compared to 7.8% in the sham group over 12 months (Danzig et al., 2024a). Both pegaptanib and avacincaptad were developed by systematic evolution of ligands by exponential enrichment (SELEX) which screens large pools of nucleotide sequences for affinity to a specific target, identifying the highest-affinity oligonucleotide ligands in many subsequent rounds of enrichment (Tuerk and Gold, 1990).

Avacincaptad pegol is a 39 nucleotide RNA aptamer with extensive chemical modifications that are heavily inspired by the success of pegaptanib. Similarly, all present pyrimidines are 2′-deoxy-2′-fluoro nucleosides, the 3′terminal end is capped with an inverted deoxythymidine bound via 3′–3′ phosphodiester linkage, and the 5′end is bound to a 43 kDa PEG moiety via aminohexyl linker. All purines are 2′-*O*-methyl substituted, with the exception of two unmodified guanosines (positions 5 and 17) and one unmodified adenosine (position 33) (Table 1) (U.S. Food and Drug Administration, 2025d). Echoing the strategy of pegaptanib, these structural features provide nuclease resistance and extend avacincaptad pegol’s half-life to support monthly dosing. Dose adjustment is similarly not required for renal or hepatic impairment, but treatment was associated with increased rates of neurovascular (wet) AMD (U.S. Food and Drug Administration, 2025d).

## 4 ASO drugs

Fomivirsen (Vitravene), an ASO indicated for the local intravitreal treatment of cytomegalovirus (CMV) retinitis in patients with acquired immunodeficiency syndrome, became the first FDA-approved RNA therapeutic in 1998 (Table 2) (Roehr, 1998; U.S. Food and Drug Administration, 1998). CMV retinitis is an opportunistic infection characterized by formation of lesions in the retina that progress to loss of vision. Fomivirsen binds to its target, human CMV immediate early region 2 (IE2) mRNA, via complementary base pairing and prevents translation of several proteins critical to viral replication (Azad et al., 1993). Fomivirsen inhibits translation through antisense hybridization of target mRNA, creating a steric blockade that limits ribosomal interaction. This binding also enables RNase H-mediated degradation of target mRNA, though less efficiently than later-generation ASOs (Anderson et al., 1996). Clinical trials utilized fundus photography of the retina to assess CMV retinitis lesions and progression, with only 44% of patients in the immediate treatment group exhibiting disease progression progression compared to 70% in the group where treatment was deferred (Vitravene Study Group, 2002). Fomivirsen is a 21-mer ASO with a full phosphorothioate (PS) backbone, a modification that would become standard practice in future ASO design. PS linkages protect ASOs from nuclease degradation and greatly enhance plasma protein binding (>90%) to limit renal clearance and dramatically prolong their half-life (Srinivasan and Iversen, 1995; Geary et al., 2002; Shemesh et al., 2016). While extensive plasma protein binding allows PS ASOs to escape filtration by the kidneys, it also promotes broad tissue distribution that can increase off-target exposure and potential for associated toxicities. PS backbone modifications can also affect potency of mRNA target regulation, with high-affinity binding to plasma proteins like α-2-macroglobulin serving as a key example (Shemesh et al., 2016; Crooke et al., 2020).

**TABLE 2:** FDA-approved ASO drugs.

ASO drug *RoA*	Chemistry	Target RNA	Indication	Year approved
Fomivirsen (Vitravene®) *IVT*	21-mer ASO; PS (−) linkages; carbonate-buffered saline solution for *IVT* injection; MW = 7.122 kDa (sodium salt) 5′-dG–dC–dG–dT–dT–dT–dG–dC–dT–dC–dT–dT–dC–dT–dT–dC–dT–dT–dG–dC–dG-3′	CMV IE2 mRNA	CMV retinitis	1998
Mipomersen (Kynamro®) *SQ*	20-mer ASO gapmer (5*-10-5*); PS (−) linkages; unbuffered aqueous solution for SQ injection; MW = 7.595 kDa (sodium salt) 5′-G*–m^5^C*–m^5^C*–m^5^U*–m^5^C*–dA–dG–dT–m^5^dC–dT–dG–m^5^dC–dT–dT–m^5^dC-G*–m^5^C*–A*–m^5^C*–m^5^C*-3′	Apo B-100 mRNA	HoFH	2013
Nusinersen (Spinraza®) *IT*	18-mer ASO; PS (−) linkages; aCSF solution for *IT* injection; MW = 7.501 kDa (sodium salt) 5′-m^5^U*–m^5^C*–A*–m^5^C*–m^5^U*–m^5^U*–m^5^U*–m^5^C*–A*–m^5^U*–A*–A*–m^5^U*–G*–m^5^C*–m^5^U*–G*–G*-3′	SMN2 pre-mRNA (ISS-N1)	SMA	2016
Inotersen (Tegsedi®) *SQ*	20-mer ASO gapmer (5*-10-5*); PS (−) linkages; unbuffered aqueous solution for *SQ* injection; MW = 7.601 kDa (sodium salt) 5′-m^5^U*–m^5^C*–m^5^U*–m^5^U*–G*–dG–dT–dT–dA–m^5^dC–dA–dT–dG–dA–dA–A*–m^5^U*–m^5^C*–m^5^C*–m^5^C*-3′	TTR mRNA	hATTR	2018
Eteplirsen (Exondys 51®) *IV*	30-mer PMO; PN (») linkages; PBS concentrate diluted in saline for *IV* infusion; MW = 10.306 kDa R » 5′-C » T » C » C » A » A » C » A » T » C » A » A » G » G » A » A » G » A » T » G » G » C » A » T » T » T » C » T » A » G-3 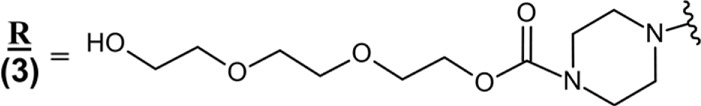	Dystrophin pre-mRNA (Exon 51)	DMD (Exon 51)	2016
Golodirsen (Vyondys 53™) *IV*	25-mer PMO; PN (») linkages; PBS concentrate diluted in saline for *IV* infusion; MW = 8.647 kDa R » 5′-G»T»T»G»C»C»T»C»C»G»G»T»T»C»T»G»A»A»G»G»T»G»T»T»C-3′	Dystrophin pre-mRNA (Exon 53)	DMD (Exon 53)	2019
Casimersen (Amondys 45™) *IV*	22-mer PMO; PN (») linkages; PBS concentrate diluted in saline for *IV* infusion; MW = 7.585 kDa R » 5′-C»A»A»T»G»C»C»A»T»C»C»T»G»G»A»G»T»T»C»C»T»G-3′	Dystrophin pre-mRNA (Exon 45)	DMD (Exon 45)	2021
Viltolarsen (Viltepso™) *IV*	21-mer PMO; PN (») linkages; saline solution for *IV* infusion; MW = 6.925 kDa 5′-C » C » T » C » C » G » G » T » T » C » T » G » A » A » G » G » T » G » T » T » C-3′	Dystrophin pre-mRNA (Exon 53)	DMD (Exon 53)	2020
Tofersen (Qalsody™) *IT*	20-mer ASO gapmer (5*-10-5*); PO (∼) and PS (−) linkages; aCSF solution for *IT* injection; MW = 7.127 kDa 5′-m^5^C*–A*∼G*–G*∼A*–dT–dA–m^5^dC–dA–dT–dT–dT–m^5^dC–dT–dA–m^5^C*∼A*–G*∼m^5^C*–m^5^U*-3′	SOD1 mRNA	SOD1-ALS	2023
Eplontersen (Wainua™) *SQ*	20-mer GalNAc-linked ASO gapmer (5*-10-5*); PO (∼) and PS (−) linkages; PBS solution for *SQ* injection; MW = 9.046 kDa (sodium salt) (GalNAc)_3_-THA∼ 5′-m^5^U*–C*∼m^5^U*∼m^5^U*∼G*∼dG–dT–dT–dA– m^5^dC–dA–dT–dG–dA–dA–A*∼m^5^U*∼m^5^C*–m^5^C*–m^5^C*-3′ 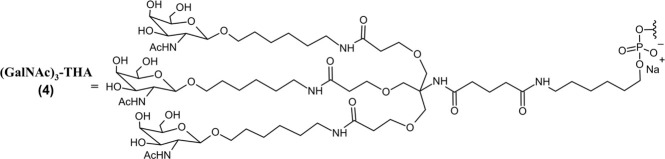	TTR mRNA	hATTR-PN	2023
Imetelstat (Rytelo™) *IV*	13-mer 3′-amino-3′-deoxy ASO; PS (−) linkages; lyophilized solid reconstituted in saline for *IV* infusion; MW = 4.896 kDa (sodium salt) R_P_ – 5′-*T*–*A*–*G*–*G*–G–*T*–*T*–*A*–*G*–*A*–*C*–*A*–*A*-NH_2_-3′ 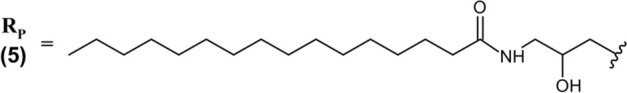	Telomerase template RNA	MDS with transfusion-dependent anemia	2024
Olezarsen (Tryngolza™) *SQ*	20-mer GalNAc-linked ASO gapmer (5*-10-5*); PO (∼) and PS (−) linkages; PBS solution for *SQ* injection; MW = 9.124 kDa (sodium salt) (GalNAc)_3_-THA∼ 5′-A*–G*–m^5^C*–m^5^U*–m^5^U*–m^5^dC–dT–dT–dG–dT–m^5^dC–m^5^dC–dA–dG–m^5^dC–m^5^U*–m^5^U*–m^5^U*–A*–m^5^U*-3′	Apo C-III mRNA	FCS	2024
Donidalorsen (Dawnzera™) *SQ*	20-mer GalNAc-linked ASO gapmer (5*-10-5*); PO (∼) and PS (−) linkages; PBS solution for *SQ* injection; MW = 9.112 kDa (sodium salt) (GalNAc)_3_-THA∼ 5′-m^5^U*–G*–m^5^C*∼A*∼A*–dG–dT–m^5^dC–dT–m^5^dC–dT–dT–dG–dG–m^5^dC–A*∼A*∼A*–m^5^C*–A*3′	PKK mRNA	HAE	2025

Abbreviations: *, 2′-*O*-(2-methoxyethyl) nucleoside; aCSF, artificial cerebrospinal fluid; Apo B-100, apolipoprotein B-100; Apo C-III, apolipoprotein C-III; ALS, amyotrophic lateral sclerosis; ASO, antisense oligonucleotide; DMD, Duchenne muscular dystrophy; GalNAc, N-acetylgalactosamine; CMV IE2, cytomegalovirus immediate early region 2; FCS, familial chylomicronemia syndrome; HAE, hereditary angioedema; hATTR, hereditary transthyretin-mediated amyloidosis; hATTR-PN, hereditary transthyretin-mediated amyloidosis with polyneuropathy; HoFH, homozygous familial hypercholesterolemia; ISS-N1, intronic splicing silencer N1; *Italicized,* 3′-amino-3′-deoxy nucleoside; *IT*, intrathecal; *IV*, intravenous; *IVT*, intravitreal; m^5^C, 5-methylcytosine; m^5^U, 5-methyluridine; MDS, myelodysplastic syndromes; PBS, phosphate buffered saline; PKK, prekallikrein PMO, phosphorodiamidate morpholino oligomer; PN (»), phosphorodiamidate linkage; PO (∼), phosphodiester linkage; PS (−), phosphorothioate linkage; *RoA*, route of administration; SMA, spinal muscular atrophy; SOD1, superoxide dismutase 1; *SQ*, subcutaneous; THA, tris-hexylamino linker; TTR, transthyretin; underlined, morpholino nucleoside; WFI, water for injection. In the absence of any other label, 2′-deoxynucleosides are assumed. Molecular weights in kDa were rounded to the nearest thousandth.

Mipomersen (Kynamro) is a 20-mer PS ASO that targets the apolipoprotein B-100 (Apo B-100) transcript, approved in 2013 to treat homozygous familial hypercholesterolemia (HoFH) via subcutaneous (SQ) injection (Table 2). HoFH is characterized by dangerous accumulation of low-density lipoprotein cholesterols (LDL-C), of which Apo B-100 is a key precursor (Raal et al., 2010; U.S. Food and Drug Administration, 2019). Mipomersen hybridizes with target Apo B-100 mRNA, preventing its translation through steric inhibition of ribosome interaction and RNase H-mediated transcript cleavage. Preclinical animal studies demonstrated reduction of liver Apo B-100 mRNA levels, as well as serum levels of Apo B-100, total cholesterol, and LDL-C (Crooke et al., 2005). Clinical assessment of mipomersen showed significant reduction of baseline LDL-C levels (36%) which served as the primary endpoint, but also demonstrated significant reduction of secondary endpoints Apo B-100 and Apolipoprotein A (Marcovina et al., 1994; McGowan et al., 2012; Santos et al., 2015). Mipomersen was the first ASO to use a “gapmer” design, featuring a central DNA gap of ten nucleotides flanked by “wings“ of five 2′-*O*-(2-methoxyethyl) (2′-MOE) modified nucleosides on each side. The 2′-MOE modified wings provide exonuclease resistance, reduce immune stimulation, and increase target affinity, while the central DNA gap enables RNase-mediated cleavage of target mRNA, a process which is otherwise inhibited by 2′-sugar modifications. Mipomersen was also the first ASO to utilize 5-methylcytosine and 5-methyluracil nucleobases, naturally occurring pyrimidine modifications that reduce immunogenicity and improve hybridization to target mRNA (Henry et al., 2000; Karikó et al., 2005; Wan and Seth, 2016; McCown et al., 2020). Mipomersen pioneered PS 2′-MOE gapmer design and would serve as a valuable example in the development of future ASO therapies. Unfortunately mipomersen received multiple black-box warnings from the FDA due to hepatotoxicity risk, with common adverse reactions including hepatic steatosis and elevations in serum transaminases like alanine aminotransferase (ALT) and aspartate aminotransferase (AST) (U.S. Food and Drug Administration, 2019). Because of these risks, mipomersen was contraindicated in patients with hepatic impairment and eventually pulled from the market in 2019 (Santos et al., 2015; Lui et al., 2021).

Nusinersen (Spinraza) is a 18-mer PS 2′-MOE ASO approved in 2016 for the treatment of spinal muscular atrophy (SMA) (Table 2) (U.S. Food and Drug Administration, 2024h). SMA is characterized by survival motor neuron (SMN) protein deficiency, leading to spinal motor neuron degeneration and death. Nusinersen works by alternative mRNA splicing, binding intronic splicing silencer N1 (ISS-N1) within the SMN2 pre-mRNA to promote exon 7 inclusion and enable production of functional SMN protein (Hua et al., 2008; Rigo et al., 2014; Chiriboga et al., 2016). In addition to the PS linkages and 2′-MOE sugar modifications mentioned, all pyrimidine nucleobases in nusinersen are 5-methyl substituted. While these modifications benefit ASO stability, potency, and tissue distribution as being discussed, they did not enable crossing of the blood-brain barrier when nusinersen was administered systemically. However, nusinersen would prove to be a great success in the treatment of SMA when administered locally via intrathecal injection.

Inotersen (Tegsedi) is a 20-mer PS ASO gapmer that was approved in 2018 for the treatment of hereditary transthyretin-mediated amyloidosis (hATTR) (Table 2), a rare genetic disorder that causes misfolding of transthyretin (TTR) protein. Misfolded TTR proteins manifest as dangerous amyloid deposits that commonly lead to cardiomyopathy and neuropathy, with kidney dysfunction also being a secondary concern. Inotersen hybridizes with TTR mRNA, promoting its degradation by RNase H. Inotersen heavily mirrors the design of mipomersen, containing the same 5-10-5 gapmer design with 2′-MOE wings, a central DNA gap, and 5-methylcytosine and 5-methyluracil nucleobases. Inotersen is similarly administered SQ and exhibits extensive plasma protein binding and widespread distribution, with the highest concentrations depositing to the liver and kidneys. Inotersen exhibited significant toxicities, receiving two black box warnings for thrombocytopenia (drastic reduction in platelet count that causes severe bleeding), and glomerulonephritis (inflammation of the kidneys, additive to the toxicity of amyloid protein buildup and consistent with severe immune response). Inotersen treatment suppresses natural vitamin A levels, explaining some of its potential toxicities and necessitating vitamin A supplementation during treatment. Overall, inotersen’s variety of potential adverse events led to very strict exclusion criteria for patient enrollment and eventually its discontinuation in 2024, with another ASO and multiple RNAi therapies for hATTR being better tolerated by patients (U.S. Food and Drug Administration, 2024i).

Eteplirsen (Exondys 51) is a 30-mer oligonucleotide that was approved in 2016 for the treatment of Duchenne muscular dystrophy (DMD) that is exon 51 amenable (Table 2). In DMD the gene encoding the dystrophin protein is not functional, with several different possible mutations capable of causing the disease. Dystrophin is a critical protein in motor function and those without a functional copy have reduced life expectancy and quality of life. Eteplirsen binds to exon 51 of dystrophin pre-mRNA, causing this exon to be skipped during mRNA splicing and resulting in a functional dystrophin protein for patients with the corresponding mutation. Eteplirsen incorporates two unique modifications, featuring uncharged phosphorodiamidate backbone linkages as well as six-membered morpholino rings replacing the natural five-membered sugar rings present in DNA/RNA. Eteplirsen was the first of four phosphorodiamidate morpholino oligonucleotides (PMOs) to be approved for DMD, followed by golodirsen (Vyondys 53) in 2019, viltolarsen (Viltepso) in 2020, and casimersen (Amondys 45) in 2021 (Table 2). All approved PMO therapies work via the same mechanism and are administered via intravenous (IV) infusion, with golodirsen and viltolarsen indicated for DMD amenable to exon 53 skipping and casimersen for DMD amenable to exon 45 skipping (Table 2) (U.S. Food and Drug Administration, 2021; U.S. Food and Drug Administration, 2024k; U.S. Food and Drug Administration, 2024c; U.S. Food and Drug Administration, 2024a). These PMO therapies’ unique structural features confer exceptional metabolic stability, each being excreted in the urine as the parent drug without any known metabolites. Distinct from PS linked ASOs, PMOs exhibit modest plasma protein binding (<40%) and are more readily cleared from the body. The PMO subclass is generally well tolerated, with renal toxicity being the primary concern. Patients with renal impairment exhibit reduced clearance of PMO drugs, but specific recommendations for dose adjustment can be challenging in DMD due to reduced muscle mass and creatinine levels.

Tofersen (Qalsody) is a 20-mer ASO gapmer with mixed PS/PO linkages, approved to treat amyotrophic lateral sclerosis (ALS) with superoxide dismutase 1 (SOD1) mutation in 2023 (Table 2) (U.S. Food and Drug Administration, 2023b). Tofersen’s structure is consistent with other ASO gapmers, containing the same 5-10-5 design with 2′-MOE wings, a central DNA gap, and 5-methylcytosine and 5-methyluracil nucleobases. Tofersen is primarily metabolized by exonucleases consistent with other ASOs but is uniquely administered via intrathecal injection. Tofersen promotes degradation of SOD1 mRNA through antisense binding, preventing accumulation of plasma neurofilament light chains that promote ALS progression.

**FIGURE 2:**
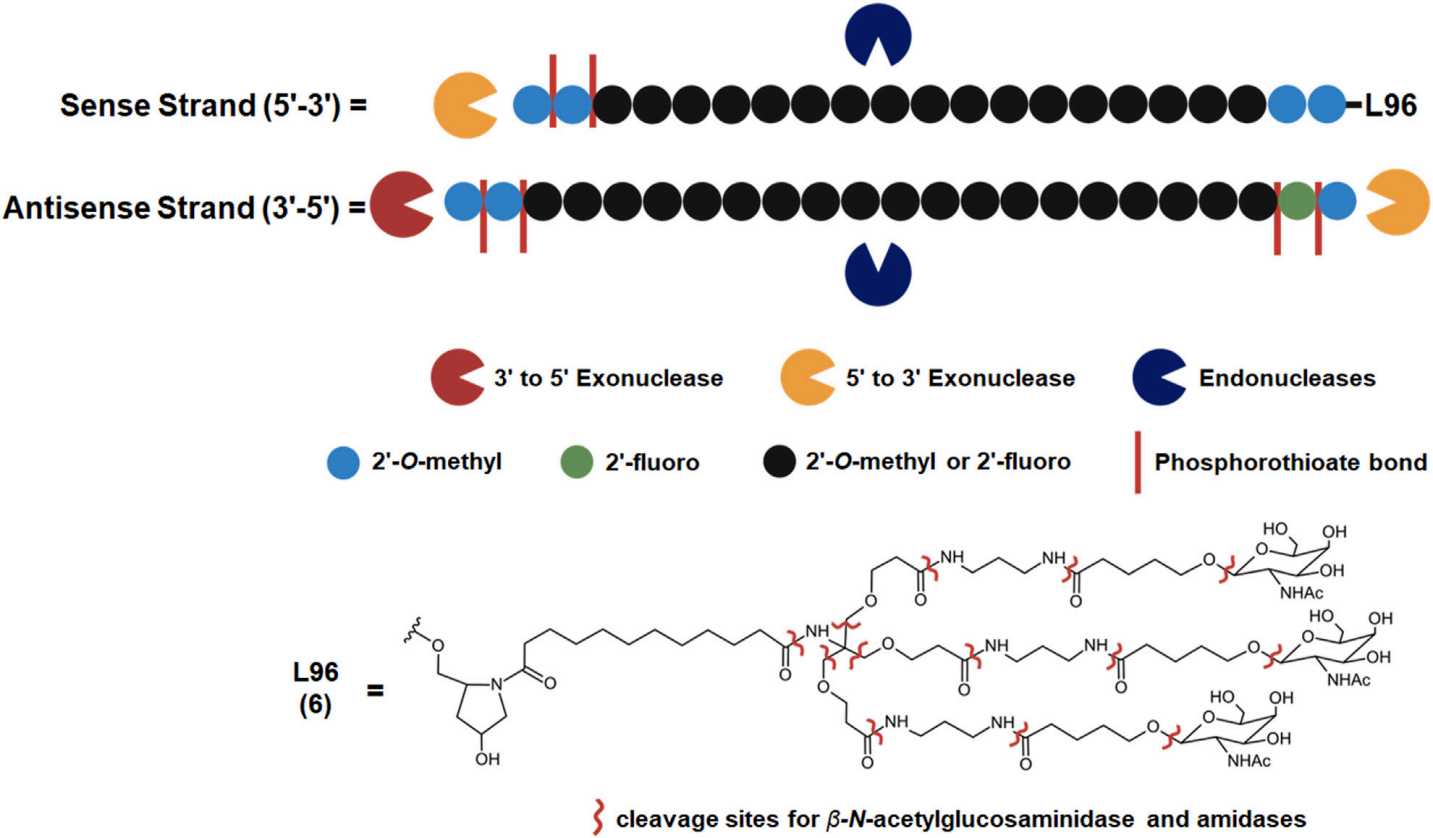
Common metabolic pathways of GalNAc-conjugated siRNA drugs. Some endogenous nucleases may cleave the sense and antisense strands at unique locations while exonucleases metabolize an RNA strand from the 5′or 3′end. Illustrated chemical modifications of ribonucleosides and the linkages are critical in reducing nuclease mediated degradation, and they are commonly found amongst the FDA-approved siRNA drugs. GalNAc conjugation of the sense 3′terminus is widely employed to improve nuclease resistance and hepatocyte uptake, found among five out of seven FDA-approved siRNA drugs. L96 and similar GalNAc moieties, however, are subject to metabolism by *ß*-*N-*acetylglucosaminidase and amidases. These enzymes can cleave several points within the L96 structure, giving rise to a variety of metabolites possessing different lengths and remaining number of GalNAc moiety.

Eplontersen (Wainua) was approved by the FDA to treat polyneuropathy of hATTR in 2023 (Table 2), and it was the first ASO drug that utilized conjugation of GalNAc ligands to achieve hepatocyte-specific delivery (Qazi et al., 2024; U.S. Food and Drug Administration, 2025h). Eplontersen is a 20-mer ASO gapmer with mixed PS/PO linkages and a 5-10-5 design consistent with other discussed therapies. Eplontersen targets the TTR transcript and promotes its degradation via RNase H following antisense hybridization. Eplontersen is conjugated to a tri-antennary GalNAc ligand (GalNAc_3_) via a trishexylamino linkage. The conjugated GalNAc moieties specifically bind to asialoglycoprotein receptors (ASGPRs) on the surface of hepatocytes, promoting ASO internalization at the target tissue. This approach has proven invaluable in the liver-targeted delivery of RNA drugs. In addition to improving target tissue specificity, GalNAc conjugation also greatly improves elimination half-lives of RNA therapies to enable less frequent dosing. Individual GalNAc ligands are cleaved by *N-*acetyl-*β*-glucosaminidase, creating several possible metabolites possessing different lengths and remaining number of GalNAc sugars (Figure 2) (Wang et al., 2019). However, GalNAc_3_-conjugated ASO is the most common form in plasma following SQ administration. Within target tissue, unconjugated ASO undergoes degradation by endo- and exonucleases consistent with the unconjugated ASOs discussed. Similarly, GalNAc-conjugated ASOs are extensively plasma protein bound, limiting their renal elimination. Multiple ASO therapies have also employed this GalNAc_3_ conjugation strategy to achieve liver-specific ASO delivery and improve tolerability of therapy (Yu R. Z. et al., 2016).

Imetelstat (Rytelo) is an ASO therapeutics possessing a unique design, approved in 2024 for the treatment of myelodysplastic syndromes (MDS) with transfusion-dependent anemia (Table 2) (U.S. Food and Drug Administration, 2024g). Imetelstat binds to the template region of Human telomerase’s RNA component. This binding elicits competitive inhibition of telomerase enzymatic activity that is overactive in malignant stem and progenitor cells and key to MDS progression (Wang et al., 2018). Imetelstat is a 13-mer ASO uniquely consisting of 3′-amino-3′deoxy nucleotides, where the natural 3′-oxygen is replaced by a nitrogen that forms a 3′-amino group. Joined with PS linkages. Imetelstat is uniquely conjugated to a palmitoyl lipid moiety that enhances its permeability and potency of telomerase inhibition (Table 2) (Herbert et al., 2005; Kazmi et al., 2019). However, IV administration of ASOs conjugated to lipophilic moieties is still often required, distinct from GalNAc conjugation that enables SQ administration (Watanabe et al., 2016).

One such therapy is Olezarsen (Tryngolza), approved in 2024 adjunct to low fat diet for familial chylomicronemia syndrome (FCS) (Table 2) (U.S. Food and Drug Administration, 2024j; Jin et al., 2025). Olezarsen targets the mRNA transcript of Apolipoprotein C-III, promoting its degradation. This improves the capability of patients to clear triglycerides and low-density cholesterols from the plasma that drive FCS progression. GalNAc_3_ conjugation is also employed in the most recent ASO drug, Donidalorsen (Dawnzera), which was approved in 2025 for the treatment of hereditary angioedema (HAE) (Table 2). Donidalorsen specifically targets prekallikrein mRNA, promoting its degradation through RNase H1. This reduces plasma prekallikrein protein levels, lowering incidence of swelling episodes characteristic of HAE (U.S. Food and Drug Administration, 2025b). GalNAc_3_-linked ASO gapmers like eplontersen, olezarsen, and donidalorsen exhibit superior tolerability and tissue specificity compared to their unconjugated predecessors and represent a significant advancement in ASO design. However, there are still limited studies assessing the use of such drugs in patients with hepatic and renal impairments, with no specific dose adjustments available based on these parameters.

## 5 RNAi drugs

Patisiran (Onpattro) represents a significant achievement in the field as the first RNAi therapy, gaining FDA approval to treat polyneuropathy of hATTR in 2018 (Table 3) (Adams et al., 2018; U.S. Food and Drug Administration, 2023a). Patisiran is a 21-mer/21-mer siRNA duplex containing an antisense strand that targets the 3′UTR of TTR mRNA with near-perfect complementarity and promotes its degradation through the RISC. Unlike later RNAi therapies, patisiran employs a lipid nanoparticle (LNP) formulation that requires administration by IV infusion. Patisiran exclusively contains natural PO backbone linkages, as well as several 2′-*O*-methyl substituted cytosine and uridine bases that limit nuclease-mediated degradation in plasma and tissues. Short chain oligonucleotide metabolites produced via nuclease degradation undergo renal elimination. Consistent with other RNA drugs, patisiran was not found to inhibit or induce major cytochrome P450 enzymes. Population studies showed no impact on the PK from mild renal and hepatic impairment, while moderate to severe impairment was not assessed.

**TABLE 3:** FDA-approved siRNA drugs.

siRNA drug *RoA*	Chemistry	Target mRNA	Indication	Year approved
Patisiran (Onpattro^™^) *IV*	21-mer/21-mer siRNA; PO (∼) linkages; LNP formulation for *IV* infusion; MW = 14.304 kDa 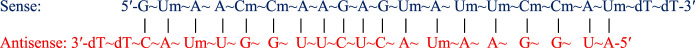	TTR	hATTR	2018
Givosiran (Givlaari^™^) *SQ*	21-mer/23-mer siRNA; only PS (−) linkages illustrated, all others PO (∼); unbuffered aqueous solution for *SQ* injection; MW = 17.246 kDa (sodium salt) 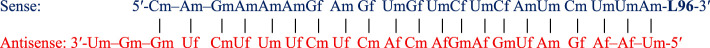 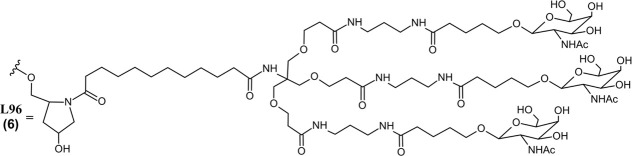	ALAS1	AHP	2019
Lumasiran (Oxlumo^™^) *SQ*	21-mer/23-mer siRNA; only PS (−) linkages illustrated, all others PO (∼); unbuffered aqueous solution for *SQ* injection; MW = 17.286 kDa (sodium salt) 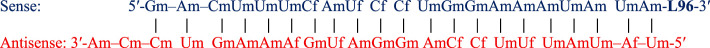	HAO1	PH1	2020
Inclisiran (Leqvio^®^) *SQ*	21-mer/23-mer siRNA; only PS (−) linkages illustrated, all others PO (∼); unbuffered aqueous solution for *SQ* injection; MW = 17.285 kDa (sodium salt) 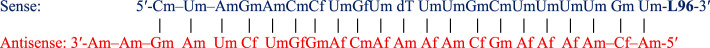	PCSK9	HeFH + ASCVD	2021
Vutrisiran (Amvuttra^®^) *SQ*	21-mer/23-mer siRNA; only PS (−) linkages illustrated, all others PO (∼); PBS solution for *SQ* injection; MW = 17.290 kDa (sodium salt) 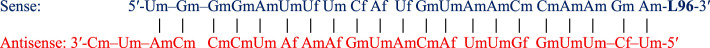	TTR	hATTR CM + PN	2022
Nedosiran (Rivfloza^®^) *SQ*	36-mer/22-mer siRNA; all PS (−) linkages illustrated, all others PO (∼); unbuffered aqueous solution for *SQ* injection; MW = 22.238 kDa (sodium salt) aG 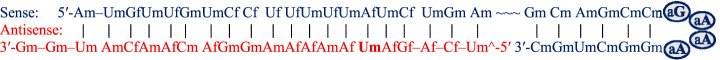 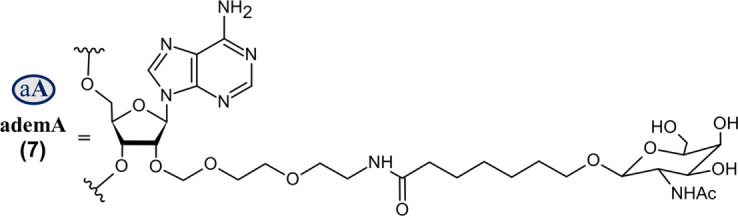 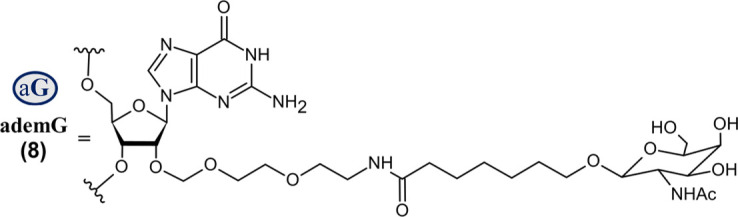	LDHA	PH1	2023
Fitusiran (Qfitlia^®^) *SQ*	21-mer/23-mer siRNA; only PS (−) linkages illustrated, all others PO (∼); PBS solution for *SQ* injection; MW = 17.193 kDa (sodium salt) 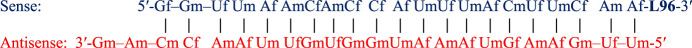	AT	Hemophilia A+ B	2025

Abbreviations: A, adenosine; AdemA, GalNAc aminosugar conjugated adenosine; AdemG, GalNAc aminosugar conjugated guanosine; Af, 2′-fluoroadenosine; AHP, acute hepatic porphyria; ALAS1, delta-aminolevulinate synthase 1; ASCVD, atherosclerotic cardiovascular disease; AT, antithrombin; CM, cardiomyopathy; Cm, 2′-*O*-methylcytidine; Gf, 2′-fluoroguanosine; Gm, 2′-*O*-methylguanosine; HAO1, hydroxyacid oxidase 1; hATTR, hereditary transthyretin-mediated amyloidosis; HeFH, heterozygous familial hypercholesterolemia; *IV*, intravenous; L96, tri-*N*-acetylgalactosamine (GalNAc); LDHA, lactate dehydrogenase A; LNP, lipid nanoparticle; PBS, phosphate buffered saline; PCSK9, proprotein convertase subtilisin/kexin type 9; PH1, primary hyperoxaluria type 1; PS (−), phosphorothioate linkage; *RoA*, route of administration; *SQ*, subcutaneous; TTR, transthyretin; Um, 2′-*O*-methyluridine; Um^, 2′-*O*-methyl-4′-*O*-((methoxy)phosphoryl)methyluridine; WFI, water for injection. Sense (blue) and antisense (red) strand sequences were obtained from FDA approved product inserts. Vertical lines illustrate Watson-Crick base pairing. Molecular weights in kDa were rounded to the nearest thousandth.

Givosiran (Givlaari), a 21-mer/23-mer siRNA to treat acute hepatic porphyria (AHP), was approved by the FDA in 2019 (U.S. Food and Drug Administration, 2024d) (Table 3). The antisense strand of givosiran targets the coding region of aminolevulinate synthase 1 (ALAS1) mRNA with near-perfect complementarity and promotes its degradation via RISC (Traber and Yu, 2024). Givosiran contains extensive 2′-fluoro and 2′-*O*-methyl substituted ribonucleosides. Givosiran’s backbone contains primarily PO linkages, with PS linkages present on the 5′terminal end of the sense strand and both terminal ends of the antisense strand (Figure 2) (Table 3) (U.S. Food and Drug Administration, 2024d). Critically, givosiran is conjugated to a tri-antennary GalNAc moiety termed L96 that provides enhanced stability and specific delivery to hepatocytes, enabling SQ administration. Within the liver, *N-*acetyl-*β*-glucosaminidase generates several possible metabolites possessing different lengths and remaining number of GalNAc sugars while nucleases generate shortened oligonucleotide sequences that can be excreted renally (Figure 2) (Agarwal et al., 2020; Li et al., 2021). The L96 ligand is conjugated to the 3′terminal end of the sense strand, improving its thermodynamic stability. This improved stability and large steric bulk provided by the L96 moiety can influence the strand-selection process, where the more thermodynamically unstable strand of the duplex is preferentially incorporated to the RISC (Khvorova et al., 2003; Schwarz et al., 2003; Gu et al., 2011). It is also shown that, when assessing RNAi drug metabolism in preclinical models, it is worth considering the impact of pH on the stability of individual sense or antisense strands, including multiple physiological pH conditions for *in vitro* testing can provide insight into how metabolism and stability may change during circulation and distribution to certain tissues and subcellular compartments (Zhang et al., 2025).

Another key structural element present in givosiran is the two-nucleotide overhang present at the antisense 3′terminus. In addition to providing further nuclease resistance, asymmetric design of siRNA duplexes influence the incorporation of the antisense/guide strand to the RISC (Sano et al., 2008). The conjugation of L96 ligand to the sense 3′terminus and presence of a two-nucleotide overhang at the 3′antisense terminus represent key advancements in the design of RNAi therapeutics that would be utilized extensively in later therapies (Figure 2). Several other siRNA drugs that mirror these structural innovations are detailed herein.

Lumasiran (Oxlumo) is a 21-mer/23-mer siRNA that was approved by the FDA in 2020 for the treatment of primary hyperoxaluria type 1 (PH1) (U.S. Food and Drug Administration, 2025e) (Table 3). The antisense strand of lumasiran targets the mRNA of hydroxyacid oxidase 1 (HAO1), hybridizing to the 3′UTR with perfect complementarity and promoting RISC-mediated degradation of the transcript (Frishberg et al., 2021; U.S. Food and Drug Administration, 2025e). Knockdown of HAO1 by lumasiran prevents harmful accumulation of calcium oxalate crystals in the urine and plasma, which are overproduced in patients with PH1 and are the underlying cause of resulting organ damage.

Inclisiran (Leqvio) is a 21-mer/23-mer siRNA that was approved by the FDA in 2021 to treat heterozygous familial hypercholesterolemia (HeFH) or existing atherosclerotic cardiovascular disease (ASCVD) (Table 3). While originally approved for use alongside statin therapy, its indication was recently expanded for use as a monotherapy (U.S. Food and Drug Administration, 2024e). Inclisiran targets the 3′UTR of the proprotein convertase subtilisin/kexin type 9 (PCSK9) transcript, binding with perfect complementarity and promoting its RISC-mediated degradation (Fitzgerald et al., 2014; Wright et al., 2020; Kallend et al., 2022; U.S. Food and Drug Administration, 2024e; Zhang et al., 2025).

Vutrisiran (Amvuttra) was approved by the FDA in 2022 to treat polyneuropathy and cardiomyopathy related to hATTR (Table 3). Vutrisiran targets the 3′UTR of the TTR transcript with perfect complementarity, promoting its degradation via RNAi (Habtemariam et al., 2021; U.S. Food and Drug Administration, 2025a). The most recent RNAi therapy to utilize this design strategy is Fitusiran (Qfitlia), which was approved by the FDA in 2025 as a prophylactic treatment for Hemophilia A and B (U.S. Food and Drug Administration, 2025f; 2025c) (Table 3). Hemophilia A and B are genetic bleeding disorders characterized by insufficient levels of pro-clotting factors VIII and IX respectively. Fitusiran aims to rebalance the coagulation cascade by suppressing antithrombin (AT), a key anticoagulant that mediates hemostasis through inhibition of several enzymes critical to clot formation, including factor IIa (thrombin), factor Xa, and factor IXa. Fitusiran hybridizes to the coding region of the antithrombin transcript with perfect complementarity, promoting its degradation to suppress AT levels and reduce bleeding risk (Sehgal et al., 2015). Clinical efficacy of fitusiran was assessed through annualized bleeding rate (ABR), with integrated analysis of multiple studies showing significant reductions in mean ABR compared to on-demand clotting factor replacement (71%), as well as on-demand and prophylactic treatment with bypassing agents (73%, 70%) (Young et al., 2025). Fitusiran has not been evaluated in a head-to-head trial against prophylactic antibody treatments like emicizumab, which similarly has a long PD half-life and exhibits sustained suppression of AT levels. However genetic or acquired deficiency of AT can increase risk of thrombotic events, highlighting the importance of an informed dosing schedule for fitusiran which was optimized to target AT activity levels between 15% and 35% based on monthly patient blood sampling (Young et al., 2025). Even with a detailed and flexible dosing strategy informed by this additional endpoint, fitusiran carries an FDA black box warning for thrombotic events as well as acute and persistent gallbladder disease (U.S. Food and Drug Administration, 2025f).

These RNAi drugs all similarly consist of a 21-mer/23-mer duplex with a 2-nucleotide antisense overhang, extensive 2′-fluoro and 2′-*O*-methyl substituted ribonucleosides, and a L96-conjugated sense strand (Figure 2A). The backbone of each duplex are primarily PO linkages, with PS linkages present on the 5′terminal end of the sense strand and both terminal ends of the antisense strand. Each of these approved siRNA therapeutics undergo some common metabolic pathways, where shortened nucleotide products are generated through endogenous nuclease cleavage (Figure 2) (An, 2024). While these GalNAc-conjugated siRNA therapies exhibit nontraditional PK/PD profiles distinct from small molecule drugs, their unique ADME properties are predictable and highly conserved across species, aiding efforts translating preclinical findings to humans (McDougall et al., 2022).

On the other hand, Nedosiran (Rivfloza), approved by the FDA in 2023 for the treatment of PH1 (Amrite et al., 2023; Goldfarb et al., 2023; U.S. Food and Drug Administration, 2025g), represents a unique strategy in siRNA design, consisting of a 36-mer/22-mer siRNA duplex (Table 3). Nedosiran contains primarily PS linkages, with a sense strand that binds itself in a hairpin-like structure. Rather than utilizing the GalNAc_3_ L96-conjugation strategy used in other approved siRNA drugs, Nedosiran uniquely employs adenosine and guanosine nucleobases that are conjugated to GalNAc sugars to enable targeted hepatocyte delivery. Nedosiran binds the 3′UTR of lactate dehydrogenase A (LDHA) mRNA, hybridizing with near-perfect complementarity to promote RISC-mediated degradation of the transcript. Knockdown of the LDHA transcript in the liver reduces lactate dehydrogenase protein levels and subsequent production of oxalate crystals in PH1 patients.

## 6 Challenges in the evaluation of DDI of RNA drugs

With diverse mechanistic actions, versatile chemistry, and distinct PK properties, RNA therapeutics provide unique strategies that have been leveraged to invaluable treatments for unaddressed diseases but also demand their own tailored strategies in assessing DDI separate from conventional approaches optimized for small molecule and protein drugs. Recent FDA guidance provides recommendations to support the development of oligonucleotide drugs, with considerations specific to this therapeutic modality including assessments of QTc interval prolongation, immunogenic risk, and possible impact of hepatic and renal impairment as well as DDIs (U.S. Food and Drug Administration, 2024b; U.S. Food and Drug Administration, 2024f). Consistent with traditional therapeutic modalities, single and multiple-dose studies are recommended to assesses the PK properties of oligonucleotide drugs in early development and determine resulting target tissue distribution, PD efficacy (e.g., reductions in target mRNA or corresponding protein, biomarker of treatment efficacy specific to disease), and DDI risk. Analytical techniques employed in determining oligonucleotide biodistribution and metabolic fate include quantitative whole body radiography (QWBA), mass-spectrometry based approaches, and stem-loop reverse transcription quantitative polymerase chain reaction (Thomas and Akoulitchev, 2006; Post et al., 2019; Wang et al., 2019; McDougall et al., 2022; Vervaeke et al., 2022). Traditional approaches in preclinical characterization of PK properties and PK-related DDI potential (e.g., assessing their potential as substrates, inhibitors, or inducers of cytochrome P450 enzymes or major drug transporters hepatocyte, recombinant, or microsomal approaches paired with probe substrates) are generally not major contributors to oligonucleotide drug metabolism, exposure, or DDI (Li et al., 2014; Shemesh et al., 2017; Ramsden et al., 2019). Oligonucleotide drugs are instead metabolized to shortened oligonucleotide chains by endogenous endo- and exonucleases that are generally well-conserved between species, reducing the likelihood of human-specific metabolites with potential for toxicity. PK and DDI assessments must consider all components of the therapeutic agent including any conjugated ligands, LNP-based delivery components, and full oligonucleotide molecule (Suzuki et al., 2023). This should also include major metabolites of examined drugs, most commonly being the parent oligonucleotide sequence shortened by 1 nucleobase, which may have hybridization-based effects of its own at the intended target or off-targets (Geary et al., 2015).

PK-based DDIs were shown uncommon across oligonucleotide drugs, with adverse events due to organ-specific PD interactions being more typical. Individual classes of RNA therapeutics (aptamers, ASOs, RNAi) and their chemical subgroups often exhibit PK properties and organ distribution very similar to one another, due in large part to the LNP or polymer formulations and ligand-conjugation strategies (e.g., GalNAc) to overcome challenges in RNA delivery and achieve preferential or targeted tissue distribution. The advancement of these delivery strategies and especially GalNAc have provided great benefit in the treatment of liver-based diseases, but there are many therapeutic targets and disease states for which this is not suitable. 1n these instances, local administration to target tissues using organ-suitable formulations (e.g. IVT administration of aptamers and fomivirsen, IT administration of nusinersen and tofersen) has been utilized. These strategies however carry their own unique challenges in assessing PK/PD relationships within the target tissue and organ-specific off target effects that contribute to DDI risk.

LNP platforms for RNA delivery are customizable, employing several different components that can be functionally optimized to preferentially target the tissue or subcellular compartment of interest. These are often lipid components, polymers, and biochemicals (PEG, lipids, cholesterol), generally well distributed to the liver and kidneys. The utility of LNP formulations extend past the RNA therapies discussed herein, with the mRNA vaccines for COVID-19 being a major example. Therapeutic investigations of miRNA mimics have also utilized LNP and extracellular vesicle-based encapsulation approaches as well as several viral expression vectors–with concern to minimize immunogenic potential in these tools (Yu et al., 2020; Traber and Yu, 2023). LNP formulations have also demonstrated their flexibility and utility in clinical trial of small activating RNA (saRNA) drug candidate that target specific transcription factors within the nucleus to promote expression of endogenous therapeutic proteins. One example is MTL-CEPBA, a saRNA that utilizes a liposomal LNP SMARTICLES formulation to achieve saRNA delivery to the nuclear compartment of target myeloid cells (Sarker et al., 2020). Customizable LNP formulations use a range of ingredients including biomolecules specific to the target of interest, synthetic polymers, and even metals. While these solutions to achieve tissue- and compartment-specific delivery of RNA drugs might enable treatment of new diseases, the variety of combined excipients utilized in these technologies highlight the importance of assessing all formulation ingredients along with the parent drug early in development to identify potential DDIs and organ-specific toxicities.

**FIGURE 3:**
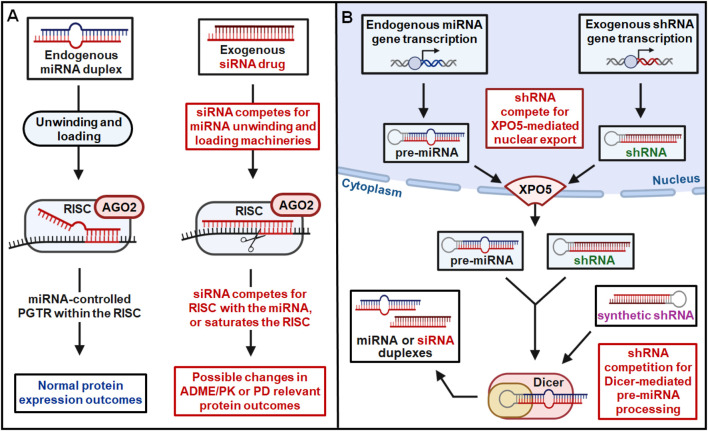
RNAi drugs may lead to risk of DDI through competition for endogenous RNAi machinery. **(A)** Competition with endogenous miRNAs for the unwinding, loading, and slicing/RISC components by siRNAs within the cytoplasm may alter the PTGR of ADME/PK or PD genes to cause possible DDI. **(B)** A shRNA, expressed within the nucleus or introduced directly into the cytoplasm, may compete with pre-miRNAs for the XPO5-mediated intracellular translocation or Dicer-mediated miRNA biogenesis, as well as the subsequent processes shown in **(A)**, leading to potential DDI.

While *in vitro* assessment of enzyme and transporter-mediated DDI is necessary for oligonucleotide drugs, those induction or inhibition studies commonly established for small molecule therapeutics may not be sufficient or suitable for RNA medications. Indeed, the clinical discovery on 2- to 3-fold increase in systemic exposure to dextromethorphan (CYP2D6) and caffeine (CYP1A2) by co-administered givosiran among subjects with acute intermittent porphyria (Vassiliou et al., 2021) was not predicted by *in vitro* findings on its lack of effects on CYP enzymes. The proposed mechanism involving the interference with hepatic heme content due to on-target ALAS1 suppression by givosiran (Vassiliou et al., 2021) cannot explain the selective impact on CYP1A2 and CYP2D6 mediated metabolism versus other CYP3A4, CYP2C9, and CYP2C19 as examined, whereas this notion seems taken in the FDA guidance (U.S. Food and Drug Administration, 2024b). Given the fact that many miRNAs have been identified to control PTGR of specific ADME genes (Yu A. M. et al., 2016; Wang et al., 2024), an inevitable competition for the RNAi machinery by siRNA drugs may significantly alter the protein levels of particular drug-metabolizing enzymes or transporters, causing possible DDIs (Figure 3) (Yu, 2007; Yu and Pan, 2012; Jilek et al., 2017; Traber et al., 2025). Likewise, potential for PD-mediated DDI and adverse events due to on-target activity should also be investigated where appropriate. One example of on-target effects leading to exaggerated PD response is fitusiran, which required additional safety assessment ahead of Phase 3 to optimize dosing schedule and mitigate risk of excessive AT suppression through using patient AT activity levels as an additional endpoint (Young et al., 2025). This additional study was critical to minimize DDI risk and provide guidance for modified dosing of on-demand factor treatments in the instance of breakthrough bleeds. This serves as an example of how oligonucleotide drugs may increase DDI risk through PD-based effect on their intended target for concomitant drugs that do not share the same target but affect the same physiological pathway.

Oligonucleotide drugs possess distinct features from other modalities and between individual classes of RNA therapies (e.g., nucleobase sequence, molecular size, chemical modifications, conjugation of varying ligands, mechanistic action) that contribute to varying tissue distribution, plasma protein binding, and potential DDI mechanisms. While aptamer drugs may assess DDI likelihood through off-target protein binding, ASO and siRNA drugs should assess potential of full or partial hybridization interactions with non-target mRNA transcripts as well as other potential endogenous DNA/RNA like mitochondrial and nuclear genomes. In particular, organ-specific off-target PD effects (e.g. miRNA-like seed hybridization at non-target transcripts by siRNA) are critical in overall safety assessments and to determine risk of DDIs with concomitant drugs. Identification and assessment of these miRNA-like interactions at non-target transcripts are routinely performed for siRNA drugs and often included in new drug application filings to the FDA prior to clinical development. It is also worth considering the impacts of RNA therapeutics across multiple organ systems, such as those targeting lipid and lipoprotein pathways (e.g., inclisiran). Modulations of lipid and lipoprotein levels across different organ systems could potentially influence the distribution and exposure of lipophilic drugs. Species differences in pharmacologically relevant tissue function and target sequence must also be addressed, with one useful approach being the use of surrogate RNA molecules possessing appropriate sequence or structure modifications that reflect species differences to allow assessment of equivalent PD endpoint in model organism. Species differences in drug effect and DDI may also be related to differences in adjacent factors within the same pathway or physiological process, as observed in non-human primate studies that over-predicted risk of complement activation from 2′MOE ASO treatment due to inhibition of factor H (Shen et al., 2014; Crooke et al., 2016). Species differences must also be considered in plasma protein binding, RNA binding proteins, and when determining ideal route of administration in nonhuman species to best recapitulate human metabolism and DDI risk (Shemesh et al., 2016).

With many RNA drugs being widely distributed and accumulating in the liver and kidneys, early preclinical and Phase 1 efforts must assess the impact of hepatic and renal impairment on PK/PD relationships and resulting DDI safety risk (Wright et al., 2020; Hoppe et al., 2022; Amrite et al., 2023; Goldfarb et al., 2023). Specifically, further assessment in renal impairment is recommended for RNA drugs that are highly cleared by the kidneys (>30%), a common characteristic of many oligonucleotide therapeutics with PS backbones that promote plasma protein binding. With prolonged PD effects and effects on organ function not always reflective of observed PK, PD-based endpoints relevant to the target organ are recommended when possible and provide valuable perspective in establishing dose-response relationships for efficacy and potential DDI (U.S. Food and Drug Administration, 2024b). Many animal species were reported to exhibit similar oligonucleotide PK properties to humans and are well suited for translational study, providing invaluable information for how target tissue PK relates to sustained PD outcomes. Population PK/PD modeling can be valuable in relating this information to organ-specific toxicity endpoints or other biomarkers to inform proper schedules and ideal doses for first-in-human studies (Geary et al., 2015; Yu R. Z. et al., 2016; Li et al., 2021; Rogers et al., 2021; McDougall et al., 2022). In general, clinical trial enrollment should emphasize patient inclusion across a substantial range of organ functions when appropriate.

Aforementioned it is critical to assess DDI risk of RNA drugs by considering the general RNAi mechanism shared by the genome derived miRNAs and exogenously introduced siRNAs (or other forms of RNAi agents), i.e., competing for the RNAi machinery that may alter endogenous miRNA function and homeostasis governing the PTGR of ADME and relevant PD genes (Figure 3). Endogenous machinery that governs the processes of miRNA duplex unwinding, RISC loading, and RNAi-based regulation may be subject to competition by exogenous RNA molecules. Natural PTGR by endogenous miRNAs is a critical determinant of protein outcomes for both PK and PD genes (Lewis et al., 2005; Yu A. M. et al., 2016; Li et al., 2019; Wang et al., 2024). By competing for critical RNAi machinery integral to RISC function such as AGO proteins, exogenous siRNA administration can disrupt natural PTGR by miRNA (Figure 3A) (Khan et al., 2009). Competition for RNAi machinery by exogenous RNAs can therefore alter the protein expression of miRNA-controlled genes to impact ADME/PK or PD function of co-administered drugs, leading to adverse DDIs (Yu and Pan, 2012; Yu A. M. et al., 2016; Jilek et al., 2017).

In addition to exogenous RNA-mediated disruption of natural PTGR by miRNA, transcribed or transfected short hairpin RNAs (shRNAs) may also contribute to potential DDI risk through competitive saturation of elements critical to endogenous miRNA biogenesis and transport (Figure 3B). One such element is exportin-5 (XPO5), a protein that mediates the nuclear export of precursor miRNAs (pre-miRNAs) and is critically important to miRNA biogenesis. Without functional XPO5 available to facilitate this transport, pre-miRNAs accumulate in the nucleus, unable to be processed to their mature miRNA duplexes by the cytoplasmic ribonuclease Dicer (Hutvagner et al., 2001; Lund and Dahlberg, 2006; Melo et al., 2010). Nuclear export by XPO5 and cytoplasmic processing by Dicer are critical steps in endogenous miRNA biogenesis, necessary for the natural PTGR of cytoplasmic miRNA targets. ShRNAs expressed within the nucleus are also substrates of XPO5, competing with endogenous pre-miRNAs for nuclear export (Figure 3B). XPO5-mediated nuclear shuttling is a rate-limiting step in endogenous miRNA biogenesis, with limited XPO5 protein being susceptible to competitive saturation by nuclear shRNA (Yi et al., 2005). Competitive saturation of XPO5 and resulting global disruption of miRNA biogenesis has significant consequences in terms of toxicities and DDI risk, demonstrated across multiple mammalian species and organ systems (Grimm et al., 2006; McBride et al., 2008; Boudreau et al., 2009; Bish et al., 2011). In addition to competitive saturation of XPO5, alteration of XPO5 activity through phosphorylation or other processes must also be considered in assessing potential toxicity and DDI risk of RNA therapies (Sun et al., 2016). In addition to these risks posed by competitive saturation or phosphorylation of XPO5, exogenous RNA molecules may also disrupt miRNA biogenesis by competing for key cytoplasmic enzyme Dicer. Many RNA molecules may be substrates of Dicer, including shRNAs exported from the nucleus as well as transfected synthetic shRNAs or siRNAs (Figure 3B). While the predominant 21-mer/23-mer duplex design strategy employed in approved RNAi therapeutics (Figure 2; Table 3) are not canonical substrates of Dicer, this is a critical factor that must be addressed when assessing DDI risk of new oligonucleotide drug candidates. Considering this critical impact on endogenous miRNA abundance and function, new RNA therapies must be assessed for potential XPO5/Dicer competition and modulation to minimize DDI risk and ensure treatment tolerability.

Assessment of DDI potential related to the disruption of endogenous small RNA pathways may also extend to other classes such as PIWI-interacting RNAs (piRNAs), which are expressed in germline cells across many animal species. This family of noncoding RNAs act in concert with PIWI proteins, impacting biological function and even drug resistance through several mechanisms including the silencing of transposable elements and epigenetic modifications. Both PIWI and AGO proteins are part of the same evolutionarily conserved family, participating in critical processes underlying the function of endogenous piRNAs, miRNAs, as well as siRNA drugs (Thomson and Lin, 2009; Wu et al., 2023). To support assessments of DDI risk due to perturbation of these pathways, further advancements in our research tools to investigate the functions and spatiotemporal characteristics of related RNA/protein complexes are needed (Hafner et al., 2021; Hazra and Spector, 2022; Hofman et al., 2025). Current approaches including cross-linking immunoprecipitation (CLIP) and fluorescence *in situ* hybridization (FISH) have been applied to interrogate ncRNA/AGO protein complexes but still carry significant challenges in spatial or temporal resolution, necessitating rigorous fractionation controls to interrogate nuclear vs. cytoplasmic interaction, for example. Additionally, UV cross-linking methods applied in CLIP and its variations may produce results that are not always representative of true cellular RNA/protein interactions, stabilizing complexes that may in fact be transient interactions. FISH methods also exhibit their own challenges, providing valuable spatial evidence for RNA but requiring concurrent immunofluorescent imaging for the protein of interest. While co-localization of these signals can provide evidence of RNA/protein proximity, this does not necessitate complex formation and can be challenging for ubiquitous proteins like AGO2 due to high background fluorescence. As sRNA therapies and their delivery strategies advance to tackle new targets and reach intracellular compartments specific to their mechanistic function, so too must our tools to interrogate the spatial and temporal aspects of these drugs and their critical partners like the AGO/PIWI family. Innovations in these methods will be critical to assess the potential impacts and DDIs of oligonucleotide drugs related to endogenous RNA pathways moving forward.

Another way oligonucleotide drugs could affect the abundance of endogenous noncoding RNAs is through disruption of target directed miRNA degradation (TDMD), which depends on AGO proteins and ubiquitin ligases like ZSWIM8 to regulate overall miRNA levels (De et al., 2013; Hiers et al., 2024). Although siRNAs are usually protected from these pathways because they are designed for preferential AGO2 loading (Frank et al., 2010; Frank et al., 2011; Ma et al., 2014; Kingston and Bartel, 2021), therapeutic concentrations could possibly saturate these shared systems. This competition could disrupt the natural degradation of endogenous miRNA, potentially altering the expression of the many genes they regulate. While the inherent stability of AGO2-bound siRNAs benefits their duration of action, it also prolongs the potential for interference with endogenous miRNA function. Continued efforts in RNAi therapeutics must prioritize the design of candidates that minimize the risk of pathway competition and make efforts to ensure their effects on the endogenous miRNA network do not become a source of adverse DDIs.

As such, it is recommended to assess the risk of DDI for RNA therapeutics by employing relevant preclinical model systems (e.g., human hepatocytes) to directly examine the effects of RNAi drugs on the activities of important drug-metabolizing enzymes and transporters (e.g., relevant probes or target drugs to be co-administered) as well as related therapeutic targets, or their protein levels. It is also noteworthy that the apparent impact could be suppression of ADME/PK/PD gene expression (protein levels; and thus function) instead of so called “induction” of enzymes or transporters applied to small molecule drugs. The latter is usually a result of the activation of nuclear receptors or transcription factors upon ligand binding, whereas oligonucleotides are unlikely to act on the ligand binding sites directly, with some chances to interfere with DNA binding domains theoretically. On the other hand, small molecule drugs have been shown to alter the levels of specific miRNAs in cells (e.g., streptomycin on miR-21, and methadone on miR-124) through interfering with miRNA biogenesis machinery or RNAi elements (Rodrigues et al., 2011; Bose et al., 2012), indicating the needs for evaluating possible DDIs with appropriate assays in proper preclinical models where the RNAi drugs are the victims of co-administered drugs. Rather, clinical investigations properly designed and completed would provide direct evidence regarding to what degrees the ADME/PK/PD or safety profiles of co-administered drugs are altered by RNA therapeutics, and vice versa, among patients.

## 7 Conclusions and perspectives

Fundamental scientific advancements pave the way for innovative therapeutic approaches, expanding the molecular toolkit of drug design to reach targets previously thought impossible. This is exemplified by the emergence of RNA or oligonucleotide therapeutics, which can harness our intrinsic biological machinery to manage human disease in novel ways and greatly improve the lives of underserved patient populations. With these innovations, however, also comes a new layer of complexity and many unanswered questions that must be addressed if we are to predict DDIs and ensure the safety of this promising treatment modality.

The distinct structural characteristics and functionalities of oligonucleotide drugs that make them uniquely useful therapeutic tools also present unique challenges in assessing their ADME/PK properties such as plasma protein binding, metabolic stability, biotransformation, and tissue distribution, requiring a specialized set of analytical techniques and risk assessment strategies. Traditional approaches utilized in the development of small molecule and protein drugs are not sufficient to predict DDI risk or elucidate PK/PD relationships for oligonucleotide drugs, with recent FDA guidance providing some recommendations. Valuable advancements have been made in the strategies to assess DDI risk of aptamers, ASOs, and siRNAs based on these features, as well as based on their distinct mechanisms of action and PD effects. However, an overlooked element is that RNA therapeutics would likely compete with endogenous miRNAs for the intrinsic RNAi components and thus alter the protein levels of miRNA-regulated ADME/PK/PD genes, leading to possible DDIs. This warrants more transparent communications among regulatory agencies and pharmaceutical industries along with neutral academic communities to provide further clarity in proper identification of the risks and avoid unwanted DDIs of novel RNA therapeutics with co-administered medications.

## References

[B1] Adachi T. Nakamura Y. (2019). Aptamers: a review of their chemical properties and modifications for therapeutic application. Molecules 24, 4229. 10.3390/molecules24234229 31766318 PMC6930564

[B2] Adams D. Gonzalez-Duarte A. O’riordan W. D. Yang C.-C. Ueda M. Kristen A. V. (2018). Patisiran, an RNAi therapeutic, for hereditary transthyretin amyloidosis. N. Engl. J. Med. 379, 11–21. 10.1056/NEJMoa1716153 29972753

[B3] Agarwal S. Simon A. R. Goel V. Habtemariam B. A. Clausen V. A. Kim J. B. (2020). Pharmacokinetics and pharmacodynamics of the small interfering ribonucleic acid, givosiran, in patients with acute hepatic porphyria. Clin. Pharmacol. Ther. 108, 63–72. 10.1002/cpt.1802 31994716

[B4] Amrite A. Fuentes E. Marbury T. C. Zhang S. (2023). Safety, pharmacokinetics, and exposure-response modeling of nedosiran in participants with severe chronic kidney disease. Clin. Pharmacol. Drug Dev. 12, 1164–1177. 10.1002/cpdd.1320 37605486

[B5] An G. (2024). Pharmacokinetics and pharmacodynamics of GalNAc-Conjugated siRNAs. J. Clin. Pharmacol. 64, 45–57. 10.1002/jcph.2337 37589246

[B6] Anderson K. P. Fox M. C. Brown-Driver V. Martin M. J. Azad R. F. (1996). Inhibition of human cytomegalovirus immediate-early gene expression by an antisense oligonucleotide complementary to immediate-early RNA. Antimicrob. Agents Chemother. 40, 2004–2011. 10.1128/AAC.40.9.2004 8878571 PMC163463

[B7] Azad R. F. Driver V. B. Tanaka K. Crooke R. M. Anderson K. P. (1993). Antiviral activity of a phosphorothioate oligonucleotide complementary to RNA of the human cytomegalovirus major immediate-early region. Antimicrob. Agents Chemother. 37, 1945–1954. 10.1128/AAC.37.9.1945 8239610 PMC188097

[B8] Basile A. S. Hutmacher M. Nickens D. Nielsen J. Kowalski K. Whitfield L. (2012). Population pharmacokinetics of pegaptanib in patients with neovascular, age-related macular degeneration. J. Clin. Pharmacol. 52, 1186–1199. 10.1177/0091270011412961 21947371

[B9] Biesecker G. Dihel L. Enney K. Bendele R. A. (1999). Derivation of RNA aptamer inhibitors of human complement C5. Immunopharmacology 42, 219–230. 10.1016/s0162-3109(99)00020-x 10408383

[B10] Bish L. T. Sleeper M. M. Reynolds C. Gazzara J. Withnall E. Singletary G. E. (2011). Cardiac gene transfer of short hairpin RNA directed against phospholamban effectively knocks Down gene expression but causes cellular toxicity in canines. Hum. Gene Ther. 22, 969–977. 10.1089/hum.2011.035 21542669 PMC3159526

[B11] Bose D. Jayaraj G. Suryawanshi H. Agarwala P. Pore S. K. Banerjee R. (2012). The tuberculosis drug streptomycin as a potential cancer therapeutic: inhibition of miR-21 function by directly targeting its precursor. Angew. Chem. Int. Ed. Engl. 51, 1019–1023. 10.1002/anie.201106455 22173871

[B12] Boudreau R. L. Martins I. Davidson B. L. (2009). Artificial microRNAs as siRNA shuttles: improved safety as compared to shRNAs *in vitro* and *in vivo* . Mol. Ther. 17, 169–175. 10.1038/mt.2008.231 19002161 PMC2834985

[B13] Chiriboga C. A. Swoboda K. J. Darras B. T. Iannaccone S. T. Montes J. De Vivo D. C. (2016). Results from a phase 1 study of nusinersen (ISIS-SMN(Rx)) in children with spinal muscular atrophy. Neurology 86, 890–897. 10.1212/WNL.0000000000002445 26865511 PMC4782111

[B14] Crooke R. M. Graham M. J. Lemonidis K. M. Whipple C. P. Koo S. Perera R. J. (2005). An apolipoprotein B antisense oligonucleotide lowers LDL cholesterol in hyperlipidemic mice without causing hepatic steatosis. J. Lipid Res. 46, 872–884. 10.1194/jlr.M400492-JLR200 15716585

[B15] Crooke S. T. Baker B. F. Kwoh T. J. Cheng W. Schulz D. J. Xia S. (2016). Integrated safety assessment of 2′-O-Methoxyethyl chimeric antisense oligonucleotides in NonHuman Primates and healthy human volunteers. Mol. Ther. 24, 1771–1782. 10.1038/mt.2016.136 27357629 PMC5112040

[B16] Crooke S. T. Vickers T. A. Liang X. H. (2020). Phosphorothioate modified oligonucleotide-protein interactions. Nucleic Acids Res. 48, 5235–5253. 10.1093/nar/gkaa299 32356888 PMC7261153

[B17] Danzig C. J. M. K. A. Loewenstein A. (2024b). C5 inhibitor avacincaptad pegol treatment for geographic atrophy: a comprehensive review. Immunotherapy 16, 779–790. 10.1080/1750743X.2024.2368342 39073397 PMC11457614

[B18] Danzig C. J. Khanani A. M. Kaiser P. K. Chang M. A. Kovach J. L. Lally D. R. (2024a). Vision loss reduction with avacincaptad pegol for geographic atrophy: a 12-Month post hoc analysis of the GATHER1 and GATHER2 trials. Ophthalmol. Retina 8, 1052–1060. 10.1016/j.oret.2024.04.023 38719191

[B19] De N. Young L. Lau P.-W. Meisner N.-C. Morrissey D. V. Macrae I. J. (2013). Highly complementary target RNAs promote release of guide RNAs from human Argonaute2. Mol. Cell 50, 344–355. 10.1016/j.molcel.2013.04.001 23664376 PMC3746828

[B20] Drolet D. W. Nelson J. Tucker C. E. Zack P. M. Nixon K. Bolin R. (2000). Pharmacokinetics and safety of an anti-vascular endothelial growth factor aptamer (NX1838) following injection into the vitreous humor of rhesus monkeys. Pharm. Res. 17, 1503–1510. 10.1023/a:1007657109012 11303960

[B21] Fire A. Xu S. Montgomery M. K. Kostas S. A. Driver S. E. Mello C. C. (1998). Potent and specific genetic interference by double-stranded RNA in *Caenorhabditis elegans* . Nature 391, 806–811. 10.1038/35888 9486653

[B22] Fitzgerald K. Frank-Kamenetsky M. Shulga-Morskaya S. Liebow A. Bettencourt B. R. Sutherland J. E. (2014). Effect of an RNA interference drug on the synthesis of proprotein convertase subtilisin/kexin type 9 (PCSK9) and the concentration of serum LDL cholesterol in healthy volunteers: a randomised, single-blind, placebo-controlled, phase 1 trial. Lancet 383, 60–68. 10.1016/S0140-6736(13)61914-5 24094767 PMC4387547

[B23] Frank F. Sonenberg N. Nagar B. (2010). Structural basis for 5′-nucleotide base-specific recognition of guide RNA by human AGO2. Nature 465, 818–822. 10.1038/nature09039 20505670

[B24] Frank F. Fabian M. R. Stepinski J. Jemielity J. Darzynkiewicz E. Sonenberg N. (2011). Structural analysis of 5′‐mRNA–cap interactions with the human AGO2 MID domain. EMBO Rep. 12, 415–420. 10.1038/embor.2011.48 21475248 PMC3090017

[B25] Frishberg Y. Deschenes G. Groothoff J. W. Hulton S. A. Magen D. Harambat J. (2021). Phase 1/2 study of lumasiran for treatment of primary hyperoxaluria type 1: a placebo-controlled randomized clinical trial. Clin. J. Am. Soc. Nephrol. 16, 1025–1036. 10.2215/CJN.14730920 33985991 PMC8425611

[B26] Geary R. S. Henry S. P. Grillone L. R. (2002). Fomivirsen: clinical pharmacology and potential drug interactions. Clin. Pharmacokinet. 41, 255–260. 10.2165/00003088-200241040-00002 11978144

[B27] Geary R. S. Baker B. F. Crooke S. T. (2015). Clinical and preclinical pharmacokinetics and pharmacodynamics of mipomersen (kynamro®): a second-generation antisense oligonucleotide inhibitor of apolipoprotein B. Clin. Pharmacokinet. 54, 133–146. 10.1007/s40262-014-0224-4 25559341 PMC4305106

[B28] Gebert L. F. Rebhan M. A. Crivelli S. E. Denzler R. Stoffel M. Hall J. (2014). Miravirsen (SPC3649) can inhibit the biogenesis of miR-122. Nucleic Acids Res. 42, 609–621. 10.1093/nar/gkt852 24068553 PMC3874169

[B29] Goldfarb D. S. Lieske J. C. Groothoff J. Schalk G. Russell K. Yu S. (2023). Nedosiran in primary hyperoxaluria subtype 3: results from a phase I, single-dose study (PHYOX4). Urolithiasis 51, 80. 10.1007/s00240-023-01453-3 37118061 PMC10147791

[B30] Gragoudas E. S. Adamis A. P. Cunningham E. T. Jr. Feinsod M. Guyer D. R. VEGF Inhibition Study in Ocular Neovascularization Clinical Trial Group (2004). Pegaptanib for neovascular age-related macular degeneration. N. Engl. J. Med. 351, 2805–2816. 10.1056/NEJMoa042760 15625332

[B31] Grimm D. Streetz K. L. Jopling C. L. Storm T. A. Pandey K. Davis C. R. (2006). Fatality in mice due to oversaturation of cellular microRNA/short hairpin RNA pathways. Nature 441, 537–541. 10.1038/nature04791 16724069

[B32] Gu S. Jin L. Zhang F. Huang Y. Grimm D. Rossi J. J. (2011). Thermodynamic stability of small hairpin RNAs highly influences the loading process of different Mammalian argonautes. Proc. Natl. Acad. Sci. 108, 9208–9213. 10.1073/pnas.1018023108 21576459 PMC3107324

[B33] Habtemariam B. A. Karsten V. Attarwala H. Goel V. Melch M. Clausen V. A. (2021). Single-dose pharmacokinetics and pharmacodynamics of transthyretin targeting N-acetylgalactosamine-Small interfering ribonucleic acid conjugate, vutrisiran, in healthy subjects. Clin. Pharmacol. Ther. 109, 372–382. 10.1002/cpt.1974 32599652

[B34] Hafner M. Katsantoni M. Köster T. Marks J. Mukherjee J. Staiger D. (2021). CLIP and complementary methods. Nat. Rev. Methods Prim. 1, 20. 10.1038/s43586-021-00018-1

[B35] Hazra R. Spector D. L. (2022). Simultaneous visualization of RNA transcripts and proteins in whole-mount mouse preimplantation embryos using single-molecule fluorescence *in situ* hybridization and immunofluorescence microscopy. Front. Cell Dev. Biol., 10–2022. 10.3389/fcell.2022.986261 36268512 PMC9577017

[B36] Henry S. Stecker K. Brooks D. Monteith D. Conklin B. Bennett C. F. (2000). Chemically modified oligonucleotides exhibit decreased immune stimulation in mice. J. Pharmacol. Exp. Ther. 292, 468–479. 10.1016/s0022-3565(24)35315-7 10640282

[B37] Herbert B. S. Gellert G. C. Hochreiter A. Pongracz K. Wright W. E. Zielinska D. (2005). Lipid modification of GRN163, an N3'P5' thio-phosphoramidate oligonucleotide, enhances the potency of telomerase inhibition. Oncogene 24, 5262–5268. 10.1038/sj.onc.1208760 15940257

[B38] Hiers N. M. Li T. Traugot C. M. Xie M. (2024). Target-directed microRNA degradation: mechanisms, significance, and functional implications. Wiley Interdiscip. Rev. RNA 15, e1832. 10.1002/wrna.1832 38448799 PMC11098282

[B39] Hofman C. R. Hu J. Bryl R. Tse V. Corey D. R. (2025). Nuclear Argonaute:miRNA complexes recognize target sequences within chromatin-associated RNA and silence gene expression. Nucleic Acids Res. 53, gkaf800. 10.1093/nar/gkaf800 40867050 PMC12390760

[B40] Hoppe B. Koch A. Cochat P. Garrelfs S. F. Baum M. A. Groothoff J. W. (2022). Safety, pharmacodynamics, and exposure-response modeling results from a first-in-human phase 1 study of nedosiran (PHYOX1) in primary hyperoxaluria. Kidney Int. 101, 626–634. 10.1016/j.kint.2021.08.015 34481803

[B41] Hua Y. Vickers T. A. Okunola H. L. Bennett C. F. Krainer A. R. (2008). Antisense masking of an hnRNP A1/A2 intronic splicing silencer corrects SMN2 splicing in transgenic mice. Am. J. Hum. Genet. 82, 834–848. 10.1016/j.ajhg.2008.01.014 18371932 PMC2427210

[B42] Hutvagner G. Mclachlan J. Pasquinelli A. E. Balint E. Tuschl T. Zamore P. D. (2001). A cellular function for the RNA-Interference enzyme dicer in the maturation of the let-7 small temporal RNA. Science 293, 834–838. 10.1126/science.1062961 11452083

[B43] Jaffe G. J. Westby K. Csaky K. G. Monés J. Pearlman J. A. Patel S. S. (2021). C5 inhibitor avacincaptad pegol for geographic atrophy due to age-related macular degeneration: a randomized pivotal phase 2/3 trial. Ophthalmology 128, 576–586. 10.1016/j.ophtha.2020.08.027 32882310

[B44] Jeon J. Y. Ayyar V. S. Mitra A. (2022). Pharmacokinetic and pharmacodynamic modeling of siRNA therapeutics - a minireview. Pharm. Res. 39, 1749–1759. 10.1007/s11095-022-03333-8 35819583

[B45] Jilek J. L. Tian Y. Yu A. M. (2017). Effects of MicroRNA-34a on the pharmacokinetics of cytochrome P450 probe drugs in mice. Drug Metab. Dispos. 45, 512–522. 10.1124/dmd.116.074344 28254952 PMC5399649

[B46] Jin J. Nguyen L. T. G. Tawfik S. M. Armanios B. Zhong X.-B. (2025). The ASO drug olezarsen targets familial chylomicronemia syndrome. Trends Pharmacol. Sci. 46, 814–815. 10.1016/j.tips.2025.05.007 40480844 PMC12335367

[B47] Kallend D. Stoekenbroek R. He Y. Smith P. F. Wijngaard P. (2022). Pharmacokinetics and pharmacodynamics of inclisiran, a small interfering RNA therapy, in patients with hepatic impairment. J. Clin. Lipidol. 16, 208–219. 10.1016/j.jacl.2022.01.001 35168913

[B48] Karikó K. Buckstein M. Ni H. Weissman D. (2005). Suppression of RNA recognition by toll-like receptors: the impact of nucleoside modification and the evolutionary origin of RNA. Immunity 23, 165–175. 10.1016/j.immuni.2005.06.008 16111635

[B49] Kazmi F. Sensenhauser C. Greway T. (2019). Characterization of the *in vitro* inhibitory potential of the oligonucleotide imetelstat on human cytochrome P450 enzymes with predictions of *in vivo* drug-drug interactions. Drug Metabolism Dispos. 47, 9–14. 10.1124/dmd.118.084103 30389730

[B50] Khan A. A. Betel D. Miller M. L. Sander C. Leslie C. S. Marks D. S. (2009). Transfection of small RNAs globally perturbs gene regulation by endogenous microRNAs. Nat. Biotechnol. 27, 549–555. 10.1038/nbt.1543 19465925 PMC2782465

[B51] Khvorova A. Reynolds A. Jayasena S. D. (2003). Functional siRNAs and miRNAs exhibit strand bias. Cell 115, 209–216. 10.1016/s0092-8674(03)00801-8 14567918

[B52] Kim R. (2007). Introduction, mechanism of action and rationale for anti-vascular endothelial growth factor drugs in age-related macular degeneration. Indian J. Ophthalmol. 55, 413–415. 10.4103/0301-4738.36473 17951895 PMC2635982

[B53] Kim J. Na H. Choi S.-Y. Oh E. J. Lee H. Ryu S. H. (2025). Structural mechanism of insulin receptor activation by a dimeric aptamer agonist. Exp. and Mol. Med. 57, 1506–1518. 10.1038/s12276-025-01494-1 40603733 PMC12322039

[B54] Kingston E. R. Bartel D. P. (2021). Ago2 protects drosophila siRNAs and microRNAs from target-directed degradation, even in the absence of 2'-O-methylation. Rna 27, 710–724. 10.1261/rna.078746.121 33853897 PMC8127995

[B55] Kovacevic K. D. Gilbert J. C. Jilma B. (2018). Pharmacokinetics, pharmacodynamics and safety of aptamers. Adv. Drug Deliv. Rev. 134, 36–50. 10.1016/j.addr.2018.10.008 30321620

[B56] Lee R. C. Feinbaum R. L. Ambros V. (1993). The *C. elegans* heterochronic gene lin-4 encodes small RNAs with antisense complementarity to lin-14. Cell 75, 843–854. 10.1016/0092-8674(93)90529-y 8252621

[B57] Lee J. H. Canny M. D. De Erkenez A. Krilleke D. Ng Y. S. Shima D. T. (2005). A therapeutic aptamer inhibits angiogenesis by specifically targeting the heparin binding domain of VEGF165. Proc. Natl. Acad. Sci. U. S. A. 102, 18902–18907. 10.1073/pnas.0509069102 16357200 PMC1323181

[B58] Lewis B. P. Burge C. B. Bartel D. P. (2005). Conserved seed pairing, often flanked by adenosines, indicates that thousands of human genes are MicroRNA targets. Cell 120, 15–20. 10.1016/j.cell.2004.12.035 15652477

[B59] Li Z. Hard M. L. Grundy J. S. Singh T. Von Moltke L. L. Boltje I. (2014). Lack of clinical pharmacodynamic and pharmacokinetic drug-drug interactions between warfarin and the antisense oligonucleotide mipomersen. J. Cardiovasc Pharmacol. 64, 164–171. 10.1097/FJC.0000000000000101 24691275

[B60] Li X. Tian Y. Tu M. J. Ho P. Y. Batra N. Yu A. M. (2019). Bioengineered miR-27b-3p and miR-328-3p modulate drug metabolism and disposition *via* the regulation of target ADME gene expression. Acta Pharm. Sin. B 9, 639–647. 10.1016/j.apsb.2018.12.002 31193825 PMC6543075

[B61] Li J. Liu J. Zhang X. Clausen V. Tran C. Arciprete M. (2021). Nonclinical pharmacokinetics and absorption, distribution, metabolism, and excretion of givosiran, the first approved N-Acetylgalactosamine-Conjugated RNA interference therapeutic. Drug Metab. Dispos. 49, 572–580. 10.1124/dmd.121.000381 33941543

[B62] Lui D. T. W. Lee A. C. H. Tan K. C. B. (2021). Management of familial hypercholesterolemia: current status and future perspectives. J. Endocr. Soc. 5, bvaa122. 10.1210/jendso/bvaa122 33928199 PMC8059332

[B63] Lund E. Dahlberg J. E. (2006). Substrate selectivity of exportin 5 and dicer in the biogenesis of microRNAs. Cold Spring Harb. Symp. Quant. Biol. 71, 59–66. 10.1101/sqb.2006.71.050 17381281

[B64] Ma H. Zhang J. Wu H. (2014). Designing Ago2-specific siRNA/shRNA to avoid competition with endogenous miRNAs. Mol. Ther. Nucleic Acids 3, e176. 10.1038/mtna.2014.27 25025466 PMC4121517

[B65] Marcovina S. M. Albers J. J. Kennedy H. Mei J. V. Henderson L. O. Hannon W. H. (1994). International Federation of clinical chemistry standardization project for measurements of apolipoproteins A-I and B. IV. Comparability of apolipoprotein B values by use of international reference material. Clin. Chem. 40, 586–592. 10.1093/clinchem/40.4.586 8149615

[B66] Martino M. T. D. Tagliaferri P. Tassone P. (2025). MicroRNA in cancer therapy: breakthroughs and challenges in early clinical applications. J. Exp. and Clin. Cancer Res. 44, 126. 10.1186/s13046-025-03391-x 40259326 PMC12010629

[B67] Mcbride J. L. Boudreau R. L. Harper S. Q. Staber P. D. Monteys A. M. Martins I. (2008). Artificial miRNAs mitigate shRNA-mediated toxicity in the brain: implications for the therapeutic development of RNAi. Proc. Natl. Acad. Sci. U. S. A. 105, 5868–5873. 10.1073/pnas.0801775105 18398004 PMC2311380

[B68] Mccown P. J. Ruszkowska A. Kunkler C. N. Breger K. Hulewicz J. P. Wang M. C. (2020). Naturally occurring modified ribonucleosides. Wiley Interdiscip. Rev. RNA 11, e1595. 10.1002/wrna.1595 32301288 PMC7694415

[B69] Mcdougall R. Ramsden D. Agarwal S. Agarwal S. Aluri K. Arciprete M. (2022). The nonclinical disposition and pharmacokinetic/pharmacodynamic properties of N-Acetylgalactosamine-Conjugated small interfering RNA are highly predictable and build confidence in translation to human. Drug Metab. Dispos. 50, 781–797. 10.1124/dmd.121.000428 34154993

[B70] Mcgowan M. P. Tardif J. C. Ceska R. Burgess L. J. Soran H. Gouni-Berthold I. (2012). Randomized, placebo-controlled trial of mipomersen in patients with severe hypercholesterolemia receiving maximally tolerated lipid-lowering therapy. PLoS One 7, e49006. 10.1371/journal.pone.0049006 23152839 PMC3496741

[B71] Melo S. A. Moutinho C. Ropero S. Calin G. A. Rossi S. Spizzo R. (2010). A genetic defect in Exportin-5 traps precursor MicroRNAs in the nucleus of cancer cells. Cancer Cell 18, 303–315. 10.1016/j.ccr.2010.09.007 20951941

[B72] Nozaki M. Raisler B. J. Sakurai E. Sarma J. V. Barnum S. R. Lambris J. D. (2006). Drusen complement components C3a and C5a promote choroidal neovascularization. Proc. Natl. Acad. Sci. U. S. A. 103, 2328–2333. 10.1073/pnas.0408835103 16452172 PMC1413680

[B73] Post N. Yu R. Greenlee S. Gaus H. Hurh E. Matson J. (2019). Metabolism and disposition of volanesorsen, a 2'-O-(2 methoxyethyl) antisense oligonucleotide, across species. Drug Metab. Dispos. 47, 1164–1173. 10.1124/dmd.119.087395 31350288

[B74] Qazi M. S. Tariq M. B. Farhan K. Salomon I. (2024). Eplontersen: a promising breakthrough in treating hereditary transthyretin amyloidosis-related polyneuropathy. Ann. Med. Surg. (Lond) 86, 4336–4337. 10.1097/MS9.0000000000002330 39118766 PMC11305710

[B75] Raal F. J. Santos R. D. Blom D. J. Marais A. D. Charng M. J. Cromwell W. C. (2010). Mipomersen, an apolipoprotein B synthesis inhibitor, for lowering of LDL cholesterol concentrations in patients with homozygous familial hypercholesterolaemia: a randomised, double-blind, placebo-controlled trial. Lancet 375, 998–1006. 10.1016/S0140-6736(10)60284-X 20227758

[B76] Ramsden D. Wu J. T. Zerler B. Iqbal S. Jiang J. Clausen V. (2019). *In vitro* drug-drug interaction evaluation of GalNAc conjugated siRNAs against CYP450 enzymes and transporters. Drug Metab. Dispos. 47, 1183–1194. 10.1124/dmd.119.087098 31270142

[B77] Rigo F. Chun S. J. Norris D. A. Hung G. Lee S. Matson J. (2014). Pharmacology of a central nervous system delivered 2'-O-methoxyethyl-modified survival of motor neuron splicing oligonucleotide in mice and nonhuman Primates. J. Pharmacol. Exp. Ther. 350, 46–55. 10.1124/jpet.113.212407 24784568 PMC4056267

[B78] Rodrigues A. C. Li X. Radecki L. Pan Y. Z. Winter J. C. Huang M. (2011). MicroRNA expression is differentially altered by xenobiotic drugs in different human cell lines. Biopharm. Drug Dispos. 32, 355–367. 10.1002/bdd.764 21796641 PMC3158292

[B79] Roehr B. (1998). Fomivirsen approved for CMV retinitis. J. Int. Assoc. Physicians AIDS Care 4, 14–16. 11365956

[B80] Rogers H. Adeniyi O. Ramamoorthy A. Bailey S. Pacanowski M. (2021). Clinical pharmacology studies supporting oligonucleotide therapy development: an assessment of therapies approved and in development between 2012 and 2018. Clin. Transl. Sci. 14, 468–475. 10.1111/cts.12945 33278337 PMC7993268

[B81] Ruckman J. Green L. S. Beeson J. Waugh S. Gillette W. L. Henninger D. D. (1998). 2'-Fluoropyrimidine RNA-Based aptamers to the 165-amino acid form of vascular endothelial growth factor (VEGF165). Inhibition of receptor binding and VEGF-Induced vascular permeability through interactions requiring the exon 7-encoded domain. J. Biol. Chem. 273, 20556–20567. 10.1074/jbc.273.32.20556 9685413

[B82] Ruscito A. Derosa M. C. (2016). Small-molecule binding aptamers: selection strategies, characterization, and applications. Front. Chem. 4, 14–2016. 10.3389/fchem.2016.00014 27242994 PMC4861895

[B83] Sano M. Sierant M. Miyagishi M. Nakanishi M. Takagi Y. Sutou S. (2008). Effect of asymmetric terminal structures of short RNA duplexes on the RNA interference activity and strand selection. Nucleic Acids Res. 36, 5812–5821. 10.1093/nar/gkn584 18782830 PMC2566866

[B84] Santos R. D. Duell P. B. East C. Guyton J. R. Moriarty P. M. Chin W. (2015). Long-term efficacy and safety of mipomersen in patients with familial hypercholesterolaemia: 2-Year interim results of an open-label extension. Eur. Heart J. 36, 566–575. 10.1093/eurheartj/eht549 24366918 PMC4344956

[B85] Sarker D. Plummer R. Meyer T. Sodergren M. H. Basu B. Chee C. E. (2020). MTL-CEBPA, a small activating RNA therapeutic upregulating C/EBP-α, in patients with advanced liver cancer: a First-in-Human, multicenter, open-label, phase I trial. Clin. Cancer Res. 26, 3936–3946. 10.1158/1078-0432.CCR-20-0414 32357963

[B86] Schwarz D. S. Hutv├Ígner G. Du T. Xu Z. Aronin N. Zamore P. D. (2003). Asymmetry in the assembly of the RNAi enzyme complex. Cell 115, 199–208. 10.1016/s0092-8674(03)00759-1 14567917

[B87] Sehgal A. Barros S. Ivanciu L. Cooley B. Qin J. Racie T. (2015). An RNAi therapeutic targeting antithrombin to rebalance the coagulation system and promote hemostasis in hemophilia. Nat. Med. 21, 492–497. 10.1038/nm.3847 25849132

[B88] Shemesh C. S. Yu R. Z. Gaus H. J. Seth P. P. Swayze E. E. Bennett F. C. (2016). Pharmacokinetic and pharmacodynamic investigations of ION-353382, a model antisense oligonucleotide: using Alpha-2-Macroglobulin and murinoglobulin double-knockout mice. Nucleic Acid. Ther. 26, 223–235. 10.1089/nat.2016.0607 27031383

[B89] Shemesh C. S. Yu R. Z. Warren M. S. Liu M. Jahic M. Nichols B. (2017). Assessment of the drug interaction potential of unconjugated and GalNAc(3)-Conjugated 2'-MOE-ASOs. Mol. Ther. Nucleic Acids 9, 34–47. 10.1016/j.omtn.2017.08.012 29246313 PMC5602538

[B90] Shen L. Frazer-Abel A. Reynolds P. R. Giclas P. C. Chappell A. Pangburn M. K. (2014). Mechanistic understanding for the greater sensitivity of monkeys to antisense oligonucleotide–mediated complement activation compared with humans. J. Pharmacol. Exp. Ther. 351, 709–717. 10.1124/jpet.114.219378 25301170

[B91] Srinivasan S. K. Iversen P. (1995). Review of *in vivo* pharmacokinetics and toxicology of phosphorothioate oligonucleotides. J. Clin. Lab. Anal. 9, 129–137. 10.1002/jcla.1860090210 7714665

[B92] Sun H.-L. Cui R. Zhou J. Teng K.-Y. Hsiao Y.-H. Nakanishi K. (2016). ERK activation globally downregulates miRNAs through phosphorylating Exportin-5. Cancer Cell 30, 723–736. 10.1016/j.ccell.2016.10.001 27846390 PMC5127275

[B93] Sutcliffe J. G. (1978). Nucleotide sequence of the ampicillin resistance gene of *Escherichia coli* plasmid pBR322. Proc. Natl. Acad. Sci. U. S. A. 75, 3737–3741. 10.1073/pnas.75.8.3737 358200 PMC392861

[B94] Suzuki Y. Katsurada Y. Hyodo K. (2023). Differences and similarities of the intravenously administered lipid nanoparticles in three clinical trials: potential linkage between lipid nanoparticles and extracellular vesicles. Mol. Pharm. 20, 4883–4892. 10.1021/acs.molpharmaceut.3c00547 37717247

[B95] Thomas B. Akoulitchev A. V. (2006). Mass spectrometry of RNA. Trends Biochem. Sci. 31, 173–181. 10.1016/j.tibs.2006.01.004 16483781

[B96] Thomson T. Lin H. (2009). The biogenesis and function of PIWI proteins and piRNAs: progress and prospect. Annu. Rev. Cell Dev. Biol. 25, 355–376. 10.1146/annurev.cellbio.24.110707.175327 19575643 PMC2780330

[B97] Traber G. M. Yu A. M. (2023). RNAi-Based therapeutics and novel RNA bioengineering technologies. J. Pharmacol. Exp. Ther. 384, 133–154. 10.1124/jpet.122.001234 35680378 PMC9827509

[B98] Traber G. M. Yu A.-M. (2024). The growing class of novel RNAi therapeutics. Mol. Pharmacol. 106, 13–20. 10.1124/molpharm.124.000895 38719476 PMC11187687

[B99] TraberG. M. TuM. J. GuanS. BatraN. YuA. M. (2025). Bioengineered miR-7-5p modulates non-small cell lung cancer cell metabolism to improve therapy. Mol. Pharmacol. 107, 100006. 10.1016/j.molpha.2024.100006 39919164

[B100] Tuerk C. Gold L. (1990). Systematic evolution of ligands by exponential enrichment: RNA ligands to bacteriophage T4 DNA polymerase. Science 249, 505–510. 10.1126/science.2200121 2200121

[B101] U.S. Food and Drug Administration (1998). Vitravene (Fomivirsen) prescribing information.

[B102] U.S. Food and Drug Administration (2011). Macugen (pegaptanib) prescribing information.

[B103] U.S. Food and Drug Administration (2019). Kynamro (mipomersen) prescribing information.

[B104] U.S. Food and Drug Administration (2021). Viletepso (viltolarsen) prescribing information.

[B105] U.S. Food and Drug Administration (2023a). Onpattro (patisiran) prescribing information.

[B106] U.S. Food and Drug Administration (2023b). Qalsody (tofersen) prescribing information.

[B107] U.S. Food and Drug Administration (2024a). Amondys 53 (casimersen) prescribing information.

[B108] U.S. Food and Drug Administration (2024b). Clinical pharmacology considerations for the development of Oligonucleotide therapeutics guidance for industry.

[B109] U.S. Food and Drug Administration (2024c). Exondys 51 (eteplirsen) prescribing information.

[B110] U.S. Food and Drug Administration (2024d). Givlaari (givosiran) prescribing information.

[B111] U.S. Food and Drug Administration (2024e). Leqvio (inclisiran) prescribing information.

[B112] U.S. Food and Drug Administration (2024f). Nonclinical safety assessment of oligonucleotide-based therapeutics guidance for industry.

[B113] U.S. Food and Drug Administration (2024g). Rytelo (imetelstat) prescribing information.

[B114] U.S. Food and Drug Administration (2024h). Spinraza (nusinersen) prescribing information.

[B115] U.S. Food and Drug Administration (2024i). Tegsedi (inotersen) prescribing information.

[B116] U.S. Food and Drug Administration (2024j). Tryngolza (olezarsen) prescribing information.

[B117] U.S. Food and Drug Administration (2024k). Vyondys 53 (golodirsen) prescribing information.

[B118] U.S. Food and Drug Administration (2025a). Amvuttra (vutrisiran) prescribing information.

[B119] U.S. Food and Drug Administration (2025b). Dawnzera (donidalorsen) prescribing information.

[B120] U.S. Food and Drug Administration (2025c). Integrated summary of effectiveness for fitusiran (NDA 219019).

[B121] U.S. Food and Drug Administration (2025d). Izervay (avacincaptad pegol) prescribing information.

[B122] U.S. Food and Drug Administration (2025e). Oxlumo (lumasiran) prescribing information.

[B123] U.S. Food and Drug Administration (2025f). Qfitlia (fitusiran) prescribing information.

[B124] U.S. Food and Drug Administration (2025g). Rivfloza (nedosiran) prescribing information.

[B125] U.S. Food and Drug Administration (2025h). Wainua (eplontersen) prescribing information.

[B126] Vassiliou D. Sardh E. Harper P. Simon A. R. Clausen V. A. Najafian N. (2021). A drug-drug interaction Study evaluating the effect of givosiran, a small interfering ribonucleic acid, on cytochrome P450 activity in the liver. Clin. Pharmacol. Ther. 110, 1250–1260. 10.1002/cpt.2419 34510420

[B127] Vervaeke P. Borgos S. E. Sanders N. N. Combes F. (2022). Regulatory guidelines and preclinical tools to study the biodistribution of RNA therapeutics. Adv. Drug Deliv. Rev. 184, 114236. 10.1016/j.addr.2022.114236 35351470 PMC8957368

[B128] Vitravene Study Group (2002). A randomized controlled clinical trial of intravitreous fomivirsen for treatment of newly diagnosed peripheral cytomegalovirus retinitis in patients with AIDS. Am. J. Ophthalmol. 133, 467–474. 10.1016/s0002-9394(02)01327-2 11931780

[B129] Wan W. B. Seth P. P. (2016). The medicinal chemistry of therapeutic oligonucleotides. J. Med. Chem. 59, 9645–9667. 10.1021/acs.jmedchem.6b00551 27434100

[B130] Wang X. Hu C. S. Petersen B. Qiu J. Ye F. Houldsworth J. (2018). Imetelstat, a telomerase inhibitor, is capable of depleting myelofibrosis stem and progenitor cells. Blood Adv. 2, 2378–2388. 10.1182/bloodadvances.2018022012 30242099 PMC6156882

[B131] Wang Y. Yu R. Z. Henry S. Geary R. S. (2019). Pharmacokinetics and clinical pharmacology considerations of GalNAc(3)-Conjugated antisense oligonucleotides. Expert Opin. Drug Metab. Toxicol. 15, 475–485. 10.1080/17425255.2019.1621838 31144994

[B132] Wang Y. Tu M.-J. Yu A.-M. (2024). Efflux ABC transporters in drug disposition and their posttranscriptional gene regulation by microRNAs. Front. Pharmacol. 15, 1423416. 10.3389/fphar.2024.1423416 39114355 PMC11303158

[B133] Watanabe A. Nakajima M. Kasuya T. Onishi R. Kitade N. Mayumi K. (2016). Comparative characterization of hepatic distribution and mRNA reduction of antisense oligonucleotides conjugated with triantennary N-Acetyl galactosamine and lipophilic ligands targeting apolipoprotein B. J. Pharmacol. Exp. Ther. 357, 320–330. 10.1124/jpet.115.230300 26907624

[B134] Wightman B. Ha I. Ruvkun G. (1993). Posttranscriptional regulation of the heterochronic gene lin-14 by lin-4 mediates temporal pattern formation in *C. elegans* . Cell 75, 855–862. 10.1016/0092-8674(93)90530-4 8252622

[B135] Wright R. S. Collins M. G. Stoekenbroek R. M. Robson R. Wijngaard P. L. J. Landmesser U. (2020). Effects of renal impairment on the pharmacokinetics, efficacy, and safety of inclisiran: an analysis of the ORION-7 and ORION-1 studies. Mayo Clin. Proc. 95, 77–89. 10.1016/j.mayocp.2019.08.021 31630870

[B136] Wu Z. Yu X. Zhang S. He Y. Guo W. (2023). Novel roles of PIWI proteins and PIWI-Interacting RNAs in human health and diseases. Cell Commun. Signal. 21, 343. 10.1186/s12964-023-01368-x 38031146 PMC10685540

[B137] Yi R. Doehle B. P. Qin Y. Macara I. G. Cullen B. R. (2005). Overexpression of exportin 5 enhances RNA interference mediated by short hairpin RNAs and microRNAs. Rna 11, 220–226. 10.1261/rna.7233305 15613540 PMC1370710

[B138] YoungG. KavakliK. KlamrothR. MatsushitaT. PeyvandiF. PipeS. W. (2025). Safety and efficacy of a fitusiran antithrombin-based dose regimen in people with hemophilia A or B: the ATLAS-OLE study. Blood 145, 2966–2977. 10.1182/blood.2024027008 40053895

[B139] Yu A. M. (2007). Small interfering RNA in drug metabolism and transport. Curr. Drug Metab. 8, 700–708. 10.2174/138920007782109751 17979658

[B140] Yu A. M. Pan Y. Z. (2012). Noncoding microRNAs: small RNAs play a big role in regulation of ADME? Acta Pharm. Sin. B 2, 93–101. 10.1016/j.apsb.2012.02.011 32154096 PMC7061715

[B141] Yu A.-M. Tu M.-J. (2022). Deliver the promise: RNAs as a new class of molecular entities for therapy and vaccination. Pharmacol. and Ther. 230, 107967. 10.1016/j.pharmthera.2021.107967 34403681 PMC9477512

[B142] Yu A. M. Tian Y. Tu M. J. Ho P. Y. Jilek J. L. (2016a). MicroRNA pharmacoepigenetics: posttranscriptional regulation mechanisms behind variable drug disposition and strategy to develop more effective therapy. Drug Metab. Dispos. 44, 308–319. 10.1124/dmd.115.067470 26566807 PMC4767381

[B143] Yu R. Z. Gunawan R. Post N. Zanardi T. Hall S. Burkey J. (2016b). Disposition and pharmacokinetics of a GalNAc3-Conjugated antisense oligonucleotide targeting human lipoprotein (a) in monkeys. Nucleic Acid. Ther. 26, 372–380. 10.1089/nat.2016.0623 27500733

[B144] Yu A. M. Choi Y. H. Tu M. J. (2020). RNA drugs and RNA targets for small molecules: principles, progress, and challenges. Pharmacol. Rev. 72, 862–898. 10.1124/pr.120.019554 32929000 PMC7495341

[B145] Yunn N. O. Lee J. Lee H. S. Oh E. J. Park M. Park S. (2022). An aptamer agonist of the insulin receptor acts as a positive or negative allosteric modulator, depending on its concentration. Exp. Mol. Med. 54, 531–541. 10.1038/s12276-022-00760-w 35478209 PMC9076861

[B146] Zamore P. D. Tuschl T. Sharp P. A. Bartel D. P. (2000). RNAi: double-stranded RNA directs the ATP-dependent cleavage of mRNA at 21 to 23 nucleotide intervals. Cell 101, 25–33. 10.1016/S0092-8674(00)80620-0 10778853

[B147] Zhang H. Qin G. Shi L. Li R. Shen L. Cao W. (2025). Comparative metabolism of an N-acetylgalactosamine–conjugated small interfering RNA, inclisiran, among various *in vitro* systems and correlations with *in vivo* metabolism in rats. Drug Metabolism Dispos. 53, 100089. 10.1016/j.dmd.2025.100089 40505270

